# Effect of artificial or autologous coverage of the pancreatic remnant or anastomosis on postoperative pancreatic fistulas after partial pancreatectomy: meta-analysis of randomized clinical trials

**DOI:** 10.1093/bjsopen/zrae059

**Published:** 2024-05-30

**Authors:** Jonas K Walber, Pia Antony, Hendrik Strothmann, Eva Kalkum, Pietro Renzulli, Fabian Hauswirth, Pascal Probst, Markus K Muller

**Affiliations:** Department of Surgery, Cantonal Hospital Thurgau, Münsterlingen, Switzerland; Department of Surgery, Cantonal Hospital Thurgau, Münsterlingen, Switzerland; Department of Surgery, Cantonal Hospital Thurgau, Münsterlingen, Switzerland; Study Centre of the German Society of Surgery (SDGC), University of Heidelberg, Heidelberg, Germany; Department of Surgery, Cantonal Hospital Thurgau, Münsterlingen, Switzerland; Department of Surgery, Cantonal Hospital Thurgau, Münsterlingen, Switzerland; Department of Surgery, Cantonal Hospital Thurgau, Frauenfeld, Switzerland; Department of Surgery, Cantonal Hospital Thurgau, Münsterlingen, Switzerland; Department of Surgery, Cantonal Hospital Thurgau, Frauenfeld, Switzerland

## Abstract

**Background:**

Postoperative pancreatic fistulas remain a driver of major complications after partial pancreatectomy. It is unclear whether coverage of the anastomosis or pancreatic remnant can reduce the incidence of postoperative pancreatic fistulas. The aim of this study was to evaluate the effect of autologous or artificial coverage of the pancreatic remnant or anastomosis on outcomes after partial pancreatectomy.

**Methods:**

A systematic literature search was performed using MEDLINE and the Cochrane Central Register of Controlled Trials (CENTRAL) up to March 2024. All RCTs analysing a coverage method in patients undergoing partial pancreatoduodenectomy or distal pancreatectomy were included. The primary outcome was postoperative pancreatic fistula development. Subgroup analyses for pancreatoduodenectomy or distal pancreatectomy and artificial or autologous coverage were conducted.

**Results:**

A total of 18 RCTs with 2326 patients were included. In the overall analysis, coverage decreased the incidence of postoperative pancreatic fistulas by 29% (OR 0.71, 95% c.i. 0.54 to 0.93, *P* < 0.01). This decrease was also seen in the 12 RCTs covering the remnant after distal pancreatectomy (OR 0.69, 95% c.i. 0.51 to 0.94, *P* < 0.02) and the 4 RCTs applying autologous coverage after pancreatoduodenectomy and distal pancreatectomy (OR 0.53, 95% c.i. 0.29 to 0.96, *P* < 0.04). Other subgroup analyses (artificial coverage or pancreatoduodenectomy) showed no statistically significant differences. The secondary endpoints of mortality, reoperations, and re-interventions were each affected positively by the use of coverage techniques. The certainty of evidence was very low to moderate.

**Conclusion:**

The implementation of coverage, whether artificial or autologous, is beneficial after partial pancreatectomy, especially in patients undergoing distal pancreatectomy with autologous coverage.

## Teaser

Postoperative pancreatic fistulas remain the major complication after partial pancreatectomy. All RCTs investigating the potential benefits of artificial or autologous coverage after partial pancreatectomy were analysed. Coverage techniques as a whole, but especially performed in distal pancreatectomy and the application of autologous material, reduce the incidence of postoperative pancreatic fistulas and mitigate their postoperative burden.

## Introduction

The development of a pancreatic fistula remains one of the most critical determinants of postoperative course after partial pancreatic resection^1,2^. The International Study Group on Pancreatic Surgery (ISGPS) defines a postoperative pancreatic fistula (POPF) as ‘fluid output of any measurable volume via an operatively placed drain with amylase activity greater than 3 times the upper normal serum value’. POPFs are further categorized clinically, based on their complication-specific severity (biochemical leak (formerly known as grade A POPFs), grade B POPFs, and grade C POPFs), thereby underlining their importance in postoperative morbidity and mortality. A biochemical leak does not cause any change in clinical condition. By contrast, grade B and C POPFs are clinically relevant, with grade B POPFs requiring a change in clinical management and grade C POPFs leading to reoperation, organ failure, or death^3^.

The extravasation of highly aggressive, protease-rich fluid outside of the pancreatic duct system from either a leaking pancreatoenteric anastomosis or resection margin into peripancreatic tissue or the peritoneal cavity may cause serious haemorrhage, intra-abdominal abscesses and fistulas, sepsis, or even death^4^. According to the ISGPS Evidence Map of Pancreatic Surgery (www.evidencemap.surgery), POPF incidence is about 14% (99% c.i. 12% to 17%) for pancreatoduodenectomy (PD) and 22% (99% c.i. 17% to 28%) for distal pancreatectomy (DP)^5^. Even at high-volume centres, the POPF-related mortality rate has remained at approximately 1% over the past 25 years^6^. Since then, several medical and surgical techniques have been employed to reduce POPF incidence and its sequelae. These include short- and long-acting somatostatin analogues, special postoperative diets, and changes to anastomosis type in PD or method of closure in DP^4,7,8^. To further protect the anastomosis or pancreas remnant after PD or DP, many have advocated for the application of a coverage material, such as the omentum or the falciform ligament (autologous) or topical haemostatic occlusive agents (artificial)^9–12^. However, to date, no clear consensus has been reached as to the benefits of autologous or artificial coverage^7,13,14^. A paucity of high-quality studies, inconsistencies in the reporting of complications, and a lack of data with regard to perioperative factors have hampered reliable conclusions as to the benefits of various coverage techniques^15^.

The aim of this systematic review and meta-analysis was the evaluation of potential benefits of artificial or autologous coverage of the pancreatic remnant or anastomosis after partial pancreatectomy.

## Methods

This systematic review and meta-analysis was completed using a previously registered review protocol (registered in PROSPERO, the international prospective register of systematic reviews (CRD42022379272)) and done in accordance with PRISMA guidelines and specific recommendations for systematic reviews in surgery^16,17^. The PRISMA checklist is available in the *Supplementary material*.

### Systematic literature search

A systematic literature search of the Cochrane Central Register of Controlled Trials (CENTRAL) and MEDLINE (via PubMed) was conducted for all RCTs dealing with partial pancreatic surgery using any coverage technique. The detailed search strategy is available in the *Supplementary material*. The last database search was performed on March 2024. No date or language restrictions were applied.

### Study selection

This meta-analysis included RCTs that met the PICOS criteria, independent of surgery type (PD or DP) and indication (benign, premalignant, or malignant pancreatic disease).

- **P** (patients): adult patients undergoing elective partial pancreatectomy
- **I** (intervention): autologous and artificial coverage of the pancreatic remnant or anastomosis
- **C** (control): no coverage or another kind of coverage of the pancreatic remnant or anastomosis
- **O** (outcome): predefined outcome parameters as described in the Data extraction section
- **S** (study design): RCT only

The selection of studies was conducted according to the recommendations of the Cochrane Collaboration^18^. In addition, a search for ongoing RCTs, as well as unpublished but completed RCTs, was performed^5^ to incorporate them in the analysis as results became available.

### Data extraction

The screening of titles and abstracts and the assessment of full texts were performed independently by two reviewers (P.P. and J.K.W.). A third reviewer was consulted in cases where no consensus could be reached as to whether a particular article should be included (M.K.M.). The primary outcome was POPF development. Further outcomes of interest were mortality, overall morbidity according to Clavien–Dindo, major complications (Clavien–Dindo grade III or higher)^19^, survival (1- to 5-year survival), reoperations, and re-interventions.

When possible, only POPF-related results were included in the analysis. A re-intervention was defined as the management of any gastroenterological or interventional radiological complication. Revisional surgeries were not categorized as re-interventions; they were categorized as reoperations. In addition, specific postoperative complications, including post-pancreatectomy haemorrhage, delayed gastric emptying, intra-abdominal collections, acute postoperative pancreatitis, bile leak, chyle leak, and surgical-site infection, and continuous variables, such as intraoperative blood loss, duration of surgery, and duration of hospital stay, were analysed. Finally, the ensuing costs of any necessary interventions were analysed.

### Critical appraisal

The methodological quality of all included RCTs was assessed according to the Cochrane Collaboration tool for assessing risk of bias (version 2). This included the following five different types of bias^18^: bias arising from the randomization process; bias due to deviations from intended intervention; bias due to missing outcome data; bias in measurement of the outcome; and bias in selection of the reported result.

Signalling questions were used for risk stratification into three levels: low risk; some concern; and high risk. Based on this information, an overall risk-of-bias assessment was performed. For certainty assessment, the Grading of Recommendations Assessment, Development, and Evaluation (GRADE) approach^20^ was used and for every outcome the certainty of evidence was rated as very low, low, moderate, or high.

### Statistical analysis

Data pooling and statistical analysis were performed using R with RStudio (version 4.2.2)^21^. All categorical data were analysed using the Mantel–Haenszel model and are presented as OR (95% c.i.). For continuous data, the mean difference (MD) and 95% c.i. were calculated using an inverse variance model. Continuous outcomes reported as median (range) were converted into mean(s.d.), according to Wan *et al*.^22^.

Because of the clinical heterogeneity, all analyses were performed using a random-effects model^17^. Heterogeneity among trials was assessed using the *I*^2^ test.

## Results

### Study selection

**Fig. 1:**
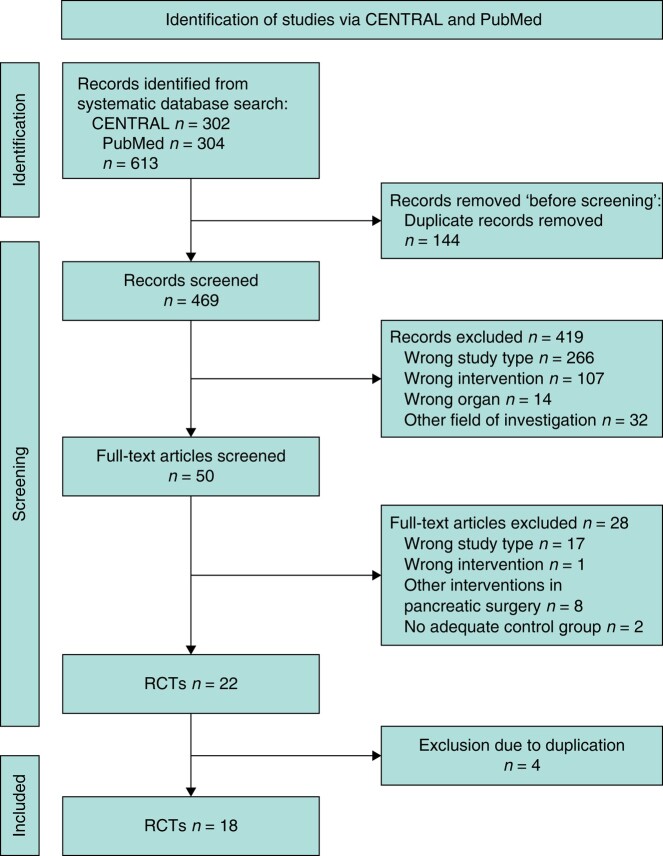
PRISMA flow chart The process of study selection according to the recommendations of the Cochrane Collaboration.

Out of 613 studies identified during the systematic literature search, 18 RCTs were included in the analysis^1,23–39^. Reasons for exclusion were wrong study type, wrong intervention, wrong organ investigated, or different field of investigation; two studies were excluded due to an inappropriate control group^40,41^ and four studies were excluded due to duplication. *Figure 1* shows the process of study selection^42^.

### Study characteristics

Of the 18 selected RCTs, which included a total of 2326 patients, 8 RCTs were multicentred^24,27–32,39^. All were published from 1994 to 2023 and were performed in 12 different countries. All studies investigated POPF development as the primary endpoint, although not all classified a POPF according to the ISGPS definition^3^; three studies diagnosed a POPF by measuring the drain leakage over a certain interval after surgery^33,34,38^, one study diagnosed a POPF using radiographic imaging^35^, and one study diagnosed a POPF using either drain leakage or radiographic imaging^26^. In PD, the anastomosis itself was covered. The vessels were not covered. In DP, the stump was covered. A total of 13 RCTs used an artificial coverage technique^23–35^, 4 RCTs applied autologous material^1,36–38^, and 1 RCT used a combination of the two^39^. A total of 11 studies used fibrin-based materials, such as patches^24,27,30–32^ and fibrin glue^25,26,33–35,39^. For autologous coverage, the ligamentum teres hepatis, the ligamentum falciforme, the omentum, and the seromuscular surface of the first jejunal loop were used. A total of 5 RCTs covered the anastomosis after PD^23–26,36^, 12 RCTs included patients after DP^1,27–35,37,38^, and 1 RCT included patients after PD and DP^39^. When a PD was performed, most anastomoses were fashioned using a double-layered duct-to-mucosa technique. For DP, the resection margin was sealed using either a stapler or manual suturing at a nearly equal rate (stapler, three RCTs; handsewn, one RCT; both techniques, seven RCTs; and not reported, two RCTs). Out of 12 studies, 7 studies performed selective duct closure in DP^1,28,31–34,37^. An internal stent of the pancreatojejunostomy was used in two studies^23,36^ and prophylactic somatostatin analogues were applied in one RCT after PD^25^ and in another RCT after DP^37^. At least 13 RCTs identified the pancreatic texture and ductal diameter as prognostic factors and stratified their analysis accordingly^23–33,36,38^. The general characteristics of the included studies are summarized in *Table 1*.

**Table 1:** Study characteristics, including basic information, as well as information on coverage material, postoperative pancreatic fistula risk factors, and surgical techniques

Reference, year (country)	*n*	PD or DP	Coverage material	Pancreas texture (hard or fibrotic/normal or soft)*		Duct diameter (mm)		Anastomosis or remnant closure (C/NC)	Prophylactic somatostatin analogues?
				C	NC	C	NC		
**Artificial coverage (*n* = 13)**									
Serradilla-Martín *et al*.^23^, 2023 (Spain)	64 (SC)	PD	Hemopatch	14/17	11/22	3.0(1.6)	3.3(2.3)	Double-layered, end-to-side duct-to-mucosa method (31/33)	NR
Schindl *et al*.^24^, 2018 (Austria)	142 (MC)	PD	TachoSil^®^ fibrin patch	29/42	34/35	≤4, 50; >4, 21	≤4, 24; >4, 47	Double-layered, end-to-side duct-to-mucosa method (71/71)	No
Martin and Au^25^, 2013 (Australia/New Zealand)	57 (SC)	PD	Tissucol/Tiseel^®^ fibrin glue	15/10	17/15	4.4(1.8)	3.9(1.5)	Blumgard anastomosis (25/32)	Yes
Lillemoe *et al*.^26^, 2004 (USA)†	124 (SC)	PD	Hemaseel APR^®^ fibrin glue	All soft		Not dilatated		Double-layered, end-to-side or end-to-end pancreatojejunostomy (58/65) Pancreatogastrostomy (0/1)	No
Mungroop *et al*.^27^, 2021 (Netherlands)	247 (MC)	DP	TachoSil^®^ fibrin patch	NR		2.3(0.8)	2(1.5)	Stapler (93/82) Handsewn (32/40)	NR
Uranues *et al*.^28^, 2021 (International)	315 (MC)	DP	Hemopatch	15/145	13/142		NR	Stapler (90/90) Handsewn (70/65) Selective duct closure (65/60)	NR
Jang *et al*.^29^, 2017 (South Korea)	97 (MC)	DP	Polyglycolic acid mesh	4/40	9/44	≤3, 40; >3, 12	≤3, 32; >3, 13	Stapler (44/53)	No
Park *et al*.^30^, 2016 (South Korea)	101 (MC)	DP	TachoSil^®^ fibrin patch	7/41	9/44	≤3, 30; >3, 18	≤3, 40; >3, 13	Stapler (Endo-GIA) (48/53)	No
Sa Cunha *et al*.^31^, 2015 (Europe)	270 (MC)	DP	TachoSil^®^ fibrin patch	7/127	12/124	NR		Stapler (54/52) Handsewn (80/84) Selective duct closure (77/72)	NR
Montorsi *et al*.^32^, 2012 (Italy)	275 (MC)	DP	TachoSil^®^ fibrin patch	23/122	16/114	≤3, 105; >3, 17	≤3, 99; >3, 15	Stapler (42/43) Handsewn (101/85) Both (1/2) NR (1/0) Selective duct closure (88/76)	No
Bassi *et al*.^33^, 1999 (Italy)†‡	40 (SC)	DP	Tissucol^®^ fibrin glue or Marlex^®^ polypropylene mesh	All soft		NR		Stapler (0/14) Handsewn (26/0) Selective duct closure (26/0)	No
Suzuki *et al*.^34^, 1995 (Japan)†	56 (SC)	DP	Tissucol/Tiseel^®^ fibrin glue	NR		NR		Handsewn (26/30) Selective duct closure (20/30)	NR
D'Andrea *et al*.^35^, 1994 (Italy)†	97 (SC)	PD and DP	Human-based fibrin glue	NR		NR		NR	NR
**Autologous coverage (*n* = 4)**									
Tangtawee *et al*.^36^, 2021 (Thailand)	68 (SC)	PD	Omentum	19/15	14/20	4(NR)	3(NR)	Double-layered, duct-to-mucosa method (31/31) Invagination (2/2) Telescopic (1/1)	NR
Hassenpflug *et al*.^1^, 2016 (Germany)	152 (SC)	DP	Teres ligament	NR		NR		Stapler (14/13) Handsewn (62/63) Selective duct closure (76/0)	NR
Oláh *et al*.^37^, 2009 (UK/Hungary)	70 (SC)	DP	Seromuscular jejunal surface	NR		NR		Stapler (35/35) Selective duct closure (27/29)	Yes
Issekutz *et al*.^38^, 2006 (Hungary)†	50 (SC)	DP	Seromuscular jejunal surface	4/50	4/51	NR		NR	NR
**Both methods for coverage (*n* = 1)**									
Carter *et al*.^39^, 2013 (USA)	109 (MC)	DP	Falciform ligament and Vitagel^®^ fibrin glue	NR		NR		Stapler (NR) Handsewn (NR)	NR

Values indicate numbers of patients or mean(s.d.). *When the study distinguished between soft, intermediate and hard pancreatic texture, intermediate was counted as hard. †Studies with a POPF definition other than that of the International Study Group on Pancreatic Surgery (Lillemoe *et al*.^26^, drainage of more than 50 ml of amylase-rich fluid per day on or after postoperative day 10 or pancreatic anastomotic disruption demonstrated radiographically; Bassi *et al*.^33^, drainage of more than 10 ml per day with an amylase content of at least 1000 U/l beyond postoperative day 7; Suzuki *et al*.^34^, drainage of pancreatic fluid for more than 7 postoperative days, diagnosed depending on local findings, such as skin excoriation around the drain site and/or smell of pancreatic secretion, and a 7-day high concentration of amylase in the drainage fluid (more than three times the serum concentration); D'Andrea *et al*.^35^, fistulas were considered only after radiological assessment; and Issekutz *et al*.^38^, drainage of more than 100 ml of fluid beyond postoperative day 5). ‡This pilot study compared five different coverage procedures of the pancreas: manual suturing; suturing plus fibrin glue; suturing plus polypropylene mesh; pancreatojejunostomy; and suturing with a stapler. In this meta-analysis, the stapler group was used as the comparison group and the mesh group and the fibrin group formed the interventional group. PD, pancreatoduodenectomy; DP, distal pancreatectomy; C, coverage; NC, no coverage; SC, single-centre study; NR, not reported; MC, multicentre study.

### Qualitative analysis—risk of bias

**Fig. 2:**
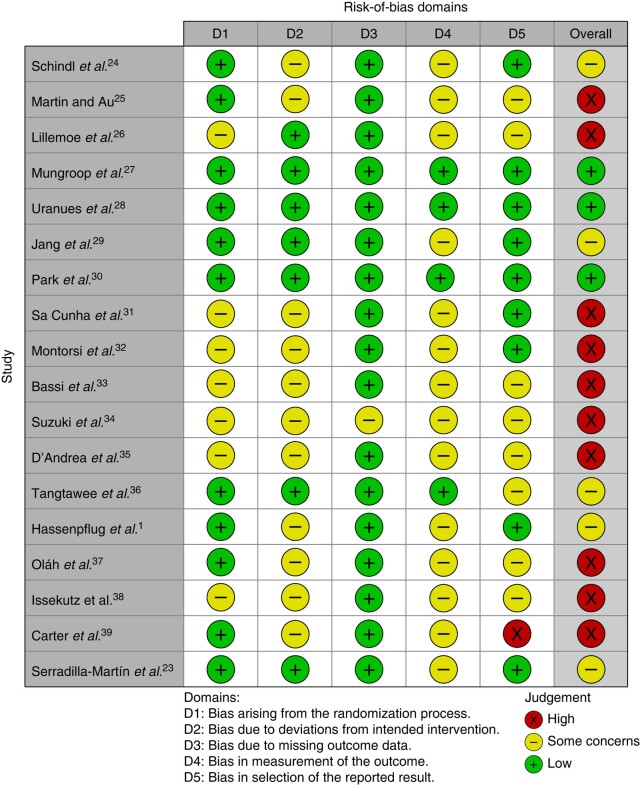
Risk-of-bias assessment The risk stratification for each study according to version 2 of the Cochrane risk-of-bias tool for randomized trials (RoB 2).

Out of the studies, ten studies were considered to be high risk for overall bias, five studies were considered to be moderate risk for overall bias, and three studies were considered to be low risk for overall bias. The main criteria for downgrading the quality grade were deviations from intended intervention and outcome measurement. In ten studies (not reported in 2 studies), the surgeon was free to choose the type of surgical anastomosis and closure technique, such as invagination, single- or double-layered anastomosis, or selective duct closure. This potentially resulted in deviations from the intended interventions by adding interventions that could influence the outcome. Most studies did not clarify to what degree the non-blinded surgeon determined the further management of the patient and thus POPF development as defined by the ISGPS. This could have potentially influenced measurement of the results. Except for Carter *et al*.^39^, none of the included studies had a bias domain that was high risk. An overview of the risk-of-bias assessment according to bias domains and overall assessment is available in *Fig. 2*.

### Quantitative analysis

Analysis was performed on all results that reached the threshold of at least three studies. Survival, chyle leak, acute postoperative pancreatitis, and costs did not reach that threshold. Subgroup analyses were performed to take coverage technique (artificial *versus* autologous) and surgical procedure (PD *versus* DP) into account. Unfortunately, subgroup analyses for many secondary endpoints were not feasible due to a lack of available data. Only subgroup results that showed a significant difference are detailed.

#### POPF

**Fig. 3:**
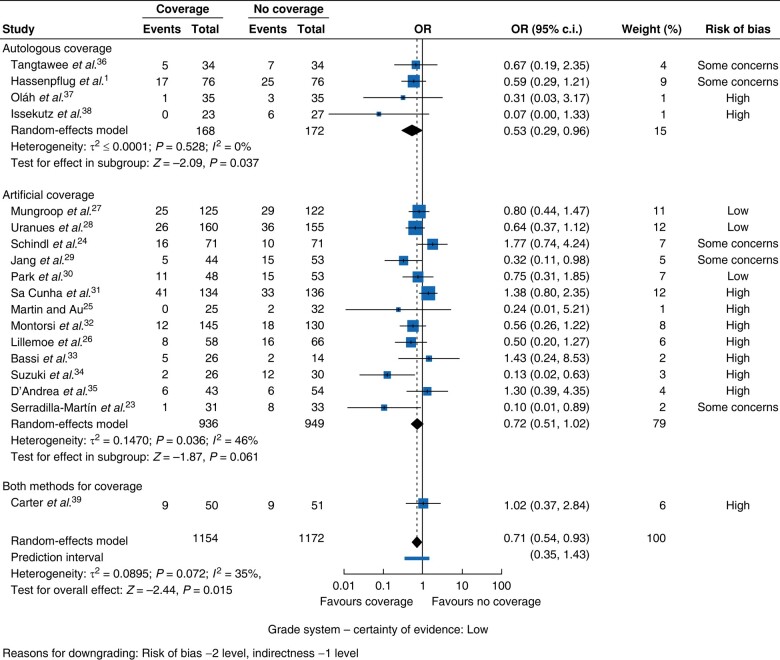
Forest plot for the ORs of all studies for postoperative pancreatic fistula rates according to type of coverage.

**Fig. 4:**
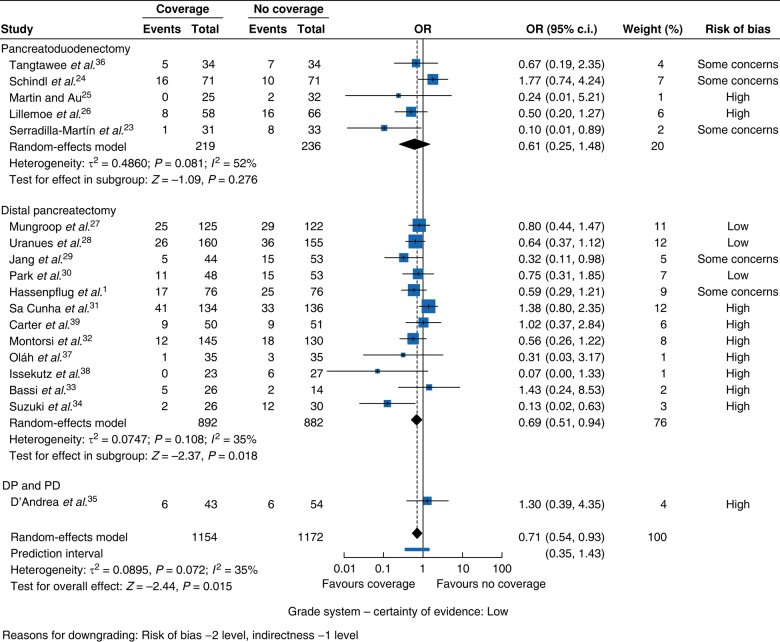
Forest plot for the ORs of all studies for postoperative pancreatic fistula rates according to type of operation DP and PD, distal pancreatectomy and pancreatoduodenectomy.

**Fig. 5:**
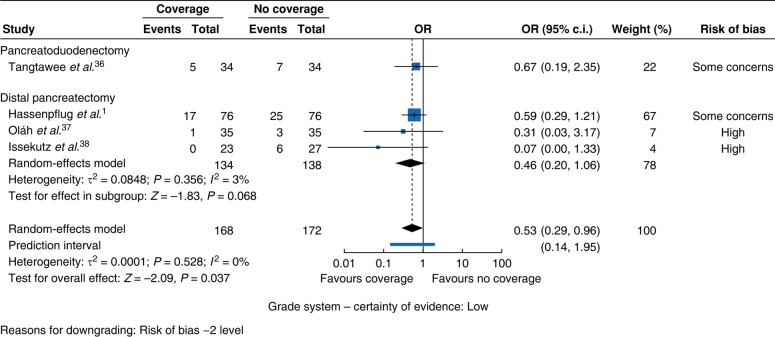
Forest plot for the ORs of studies with autologous coverage for postoperative pancreatic fistula rates.

**Fig. 6:**
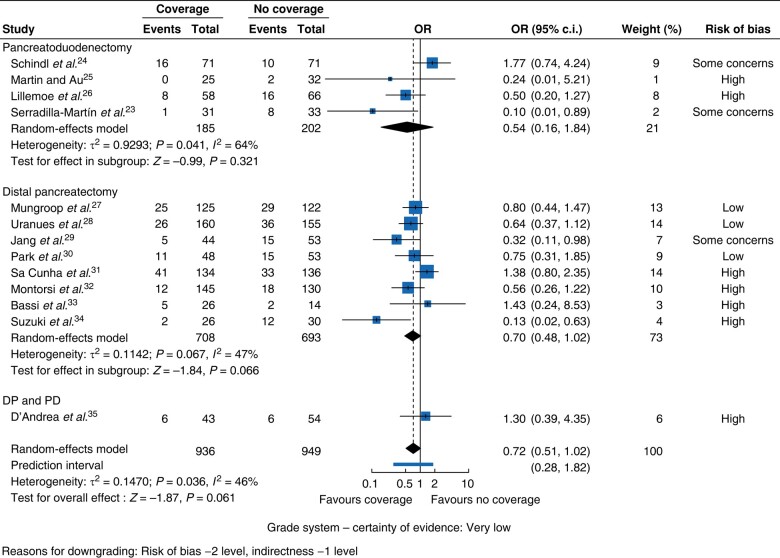
Forest plot for the ORs of studies with artificial coverage for postoperative pancreatic fistula rates DP, distal pancreatectomy; PD, pancreatoduodenectomy

In the overall analysis, the POPF rate was significantly lower in the coverage group compared with the non-coverage group (OR 0.71, 95% c.i. 0.54 to 0.93, *P* = 0.015, *I*^2^ = 35%, GRADE = low) (*Fig. 3*). POPF reduction was observed in DP regardless of the type of coverage applied (OR 0.69, 95% c.i. 0.51 to 0.94, *P* = 0.018, *I*^2^ = 35%, GRADE = low) (*Fig. 4*) and, when using autologous coverage, independent of surgery type (OR 0.53, 95% c.i. 0.29 to 0.96, *P* = 0.037, *I*^2^ = 0%, GRADE = moderate) (*Fig. 5*). No POPF reduction was observed when applying only artificial coverage in PD or DP (*Fig. 6*).

A sensitivity analysis was performed for clinically relevant POPFs (grade B/C POPFs) as the outcome. From 13 of the 18 studies that defined a POPF according to the ISGPS definition, only the clinically relevant cases were included. The remaining five RCTs did not explicitly distinguish between biochemical leak and clinically relevant POPFs (grade B/C POPFs)^26,33–35,38^. Fortunately, in two of these five studies, the cases that were clinically relevant could be identified and included in the sensitivity analysis^26,38^. Finally, 15 studies were analysed. The sensitivity analysis also showed a significant decrease in the occurrence of clinically relevant POPFs in the overall analysis (OR 0.72, 95% c.i. 0.54 to 0.95, *P* = 0.020, *I*^2^ = 30%, GRADE = low) and similar results in subgroup analyses compared with the main analyses (*Figs S1–S4*).

#### Mortality

The mortality rate was reported in 17 RCTs. A total of 6 patients (0.5%) died in the coverage group, whereas 19 patients (1.7%) died in the non-coverage group, showing a statistically significant difference and an advantage for the implementation of the different coverage techniques (OR 0.38, 95% c.i. 0.17 to 0.88, *P* = 0.025, *I*^2^ = 0%, GRADE = low) (*Fig. S5*). No statistically significant differences were observed in subgroup analyses (DP, PD, autologous, or artificial) for mortality (*Figs S5–S8*).

#### Overall morbidity

No significant difference was observed with regard to postoperative morbidity in the whole cohort (OR 0.87, 95% c.i. 0.50 to 1.51, *P* = 0.621, *I*^2^ = 16%, GRADE = low) or in subgroups of DP or PD (*Fig. S9*).

#### Complications, Clavien–Dindo grade III or higher

No significant difference was observed with regard to postoperative morbidity of Clavien–Dindo grade III or higher in the whole cohort (OR 0.89, 95% c.i. 0.64 to 1.24, *P* = 0.503, *I*^2^ = 0%, GRADE = moderate) or in subgroups of DP or PD (*Fig. S10*).

#### Reoperations and re-interventions

The reoperation rates were significantly lower in patients with coverage than in patients without coverage (OR 0.45, 95% c.i. 0.26 to 0.77, *P* < 0.004, *I*^2^ = 0%, GRADE = low) (*Fig. S11*). A significant reduction in reoperations was also observed when the analysis was limited to studies only analysing DP (OR 0.31, 95% c.i. 0.15 to 0.65, *P* < 0.002, *I*^2^ = 0%, GRADE = moderate) (*Fig. S12*) and in studies where DP was performed with either autologous (OR 0.16, 95% c.i. 0.03 to 0.72, *P* = 0.017, *I*^2^ = 0%, GRADE = low) or artificial (OR 0.38, 95% c.i. 0.16 to 0.89, *P* = 0.026, *I*^2^ = 0%, GRADE = moderate) coverage (*Figs S13, S14*), but not in studies of PD (OR 0.68, 95% c.i. 0.31 to 1.50, *P* < 0.340, *I*^2^ = 0%, GRADE = low) (*Fig. S12*). When analysing only studies with artificial coverage, there was a significant reduction in the need for reoperations (OR 0.52, 95% c.i. 0.29 to 0.93, *P* = 0.027, *I*^2^ = 0%, GRADE = moderate) (*Fig. S13*).

The same statistically significant benefit was observed when the rate of re-interventions was compared (OR 0.61, 95% c.i. 0.39 to 0.97, *P* = 0.035, *I*^2^ = 0%, GRADE = low) (*Fig. S15*). Re-interventions were significantly less frequent in groups undergoing DP with autologous coverage (OR 0.38, 95% c.i. 0.18 to 0.78, *P* = 0.008, *I*^2^ = 0%, GRADE = low); no differences were observed in other subgroups (all PD, all DP, or artificial) (*Figs S16–S18*).

#### Post-pancreatectomy haemorrhage

No difference in the rate of postoperative haemorrhage was observed in patients with coverage *versus* patients without coverage in the whole cohort (OR 0.77, 95% c.i. 0.42 to 1.38, *P* = 0.375, *I*^2^ = 0%, GRADE = moderate) or in any subgroups (*Figs S19–S22*).

#### Other pancreatic surgery-specific complications

The rates of specific postoperative complications, such as delayed gastric emptying (OR 0.70, 95% c.i. 0.42 to 1.15, *P* = 0.154, *I*^2^ = 0%, GRADE = moderate), intra-abdominal collections (OR 1.00, 95% c.i. 0.75 to 1.33, *P* = 0.985, *I*^2^ = 0%, GRADE = moderate), surgical-site infection (OR 0.93, 95% c.i. 0.58 to 1.49, *P* = 0.770, *I*^2^ = 0%, GRADE = moderate), and bile leak (OR 0.82, 95% c.i. 0.31 to 2.21, *P* = 0.700, *I*^2^ = 0%, GRADE = very low), showed no statistically significant difference in the whole cohort or in subgroups (*Figs S23–S34*).

#### Duration of surgery

Covering the pancreas after DP or PD did not significantly affect the total operating time in the whole cohort (MD +3.91 min, 95% c.i. −19.93 min to +12.12 min, *P* = 0.633, *I*^2^ = 73%, GRADE = moderate) or in subgroups (*Figs S35–S37*).

#### Intraoperative blood loss

Coverage did not affect intraoperative blood loss in the whole cohort (MD −56.70 ml, 95% c.i. −141.52 ml to +28.12 ml, *P* = 0.190, *I*^2^ = 77%, GRADE = low), but lower intraoperative blood loss was observed in patients receiving artificial coverage (MD −40.83 ml, 95% c.i. −72.52 ml to −9.14 ml, *P* = 0.012, *I*^2^ = 45%, GRADE = moderate) (*Figs S38*–S40).

#### Duration of hospital stay

There was no difference in duration of hospital stay between the coverage and non-coverage groups in the whole cohort (MD −0.62 days, 95% c.i. −4.91 days to +2.92 days, *P* = 0.618, *I*^2^ = 97%, GRADE = very low) or in subgroups (*Figs S41*–*S44*).

### Ongoing trials

Looking at the Evidence Map of Pancreatic Surgery (www.evidencemap.surgery), five ongoing RCTs investigating the potential benefit of autologous coverage and three ongoing RCTs investigating the potential benefit of artificial coverage were found; six RCTs are analysing coverage techniques in PD and two RCTs are analysing coverage techniques in DP. An overview of the studies, including their expected dates of completion, their sample sizes, and whether or not they are stratified according to POPF risk, is available in *Table 2*.

**Table 2:** All ongoing RCTs regarding coverage, including sample size and stratification according to POPF risk

Identifier	Title	Expected end of trial	Sample size, *n*	Stratification according to POPF risk?
**Autologous coverage**				
PD				
ChiCTR2000032599	The application of omental-pad technic in prevention of severe complication caused by pancreatic fistula after pancreaticoduodenectomy: a randomised controlled trial	Not updated	200	Not reported
NCT04704882	A modified omental patch work decreases pancreatic fistula after LPD	Not updated	162	Not reported
ChiCTR2000036478	A prospective randomised controlled trial on the effect of omentum wrapped pancreaticojejunostomy on pancreatic fistula and other complications	Not reported	Not reported	Not reported
CTRI/2021/06/033991	To study the efficacy of omental roll-up technique in pancreaticojejunostomy as a strategy to prevent pancreatic fistula after pancreaticoduodenectomy	Not reported	Not reported	Not reported
** **DP				
** **NCT03752086	Greater omentum binding to the pancreatic stump to prevent pancreatic fistula following distal pancreatectomy	Not updated	200	Not reported
**Artificial coverage**				
PD				
NCT03331718	Polyglycolic acid felt reinforcement of the pancreaticojejunostomy (PLANET-PJ trial)	Not updated	514	Yes
** **NCT03419676	Use of Hemopatch as a sealant at the pancreaticojejunostomy after pancreatoduodenectomy to prevent postoperative pancreatic fistula	Not updated	64	Not reported
** **DP				
NCT03201653	A prospective randomised trial of efficacy of stump closure for distal pancreatectomy	Not updated	84	Not reported

POPF, postoperative pancreatic fistula; LPD, laparoscopic pancreatoduodenectomy.

## Discussion

The present meta-analysis included 18 RCTs to evaluate the potential benefit of covering the pancreatoenteric anastomosis or pancreatic remnant with regard to decreasing the POPF rate. The meta-analysis indicated POPF reduction when coverage was applied. Additionally, significantly lower postoperative mortality, reoperation, and re-intervention rates were seen with coverage. Furthermore, the sub-analysis showed a reduction in the POPF rate after DP regardless of the type of coverage applied and, for autologous coverage, independent of surgery type. However, the certainty of evidence for most outcomes was low to moderate due to the high risk of bias, imprecision, and inconsistency.

The RCTs included in the meta-analysis were published over an interval of nearly 30 years, highlighting the controversy regarding this topic. Different meta-analyses have shown conflicting results^7,9,10,13,14,40^, raising questions with regard to how such inconsistencies arise and how the present meta-analysis differs from other meta-analyses.

A possibility may be a limited study population, such as in Tieftrunk *et al*.^7^. This was also reflected in the present data. Only 3 studies were able to show a statistically significant benefit^23,29,34^, whereas 10 studies were only able to produce a statistical trend favouring coverage^1,25–28,30,32,36–38^. With regard to mortality, the lack of power was even more noticeable. A total of 17 RCTs documented mortality rates, but only 9 RCTs reported cases of mortality^1,23,24,26–28,31,36,37^. As mortality is a rare event, larger study populations would be needed to demonstrate the potential for a reduction in mortality through coverage. The occurrence of complications after PD and DP depends on various factors, such as surgical technique and experience^10,15^. Few studies attempted to minimize such confounding factors by, for example, only using Endo-GIA staplers^31^.

Several scores have been developed to predict POPF likelihood. For PD, the alternative fistula risk score includes the risk variables of soft pancreatic texture, small duct diameter, and higher BMI^41^. For DP, the intraoperative DP fistula risk score includes neck thickness, pancreatic duct diameter, soft pancreatic texture, long operating time, and BMI as risk factors^43^. The ‘DISPAIR’ score is a competing model that identifies the pancreatic neck as the transection site, increased thickness at the transection site, and the absence of diabetes mellitus as risk factors; regarding this score, Bonsdorff *et al*.^44^ state that measurement of pancreatic thickness is more objective than assessment of glandular texture and pancreatic duct diameter. Of the analysed studies, two-thirds stratified their populations based on at least one of the mentioned risk factors. Out of the 18 studies, 2 studies took into account the idea that coverage techniques may only be advantageous in patients with a high risk of anastomosis or closure failure and only included patients with a soft pancreas. Nevertheless, both of these studies failed to show any significant benefits^35,36^, although Serradilla-Martín *et al*.^23^ found a positive correlation between a higher fistula risk score and better protection by covering the anastomosis. Further validation of risk factors for POPF occurrence is necessary, but risk scores could provide a basis for the decision to perform additional pancreatic coverage in the future.

Confounding factors could also be found in previous meta-analyses, as included studies performed additional or different closure techniques that could have interfered with the results^9,13^. In the present meta-analysis, care was taken to include only RCTs with autologous or artificial coverage as the only intervention; furthermore, only RCTs were included in the present meta-analysis. In contrast, previous meta-analyses obtained most of their data from retrospective, non-randomized studies^7,9,10,12,40^. Like the limited study populations in previous RCTs, the power of former meta-analyses was reduced by a low number of RCTs. This was mostly due to the analysis of specific coverage materials, such as fibrin glue or polyglycolic acid mesh^7,9,11,40^. The present meta-analysis performed a group analysis of all previous RCTs investigating artificial and autologous coverage techniques for PD and DP to determine, whether coverage techniques as a whole are feasible and safe in the case of PD and DP. The detailed subgroup analysis was not restricted to a specific material, so that more RCTs per subgroup could be analysed than in previous studies, including the most recent one by Serradilla-Martín *et al*.^23^, to evaluate with a higher power whether artificial or autologous coverage of the pancreatic remnant or anastomosis after partial pancreatectomy shows a potential benefit. The cumulation of the study populations in this meta-analysis reveals the risk-reducing effect of coverage procedures.

Another possible explanation for the observed inconsistencies in the analysed RCTs may lie in the different POPF definitions. Tan *et al*.^45^ claimed that POPF incidence increased from 15% to 39% when the ISGPF definition was applied. To address this issue, a sensitivity analysis, including only clinically relevant POPFs, was performed and the advantages of POPF prevention with coverage techniques remained the same.

The overall POPF rates of the present meta-analysis were comparable to the benchmark rates of the ISGPS Evidence Map of Pancreatic Surgery for PD (16.0% *versus* 14% respectively) and DP (20.1% *versus* 22% respectively)^5^. Regarding the sub-analyses, it must be noted that a possible positive effect of coverage was seen only in DP and not in PD. This fact was already postulated in other analyses and could be explained by a higher POPF incidence after DP^46^. The reason for this higher incidence lies in the different pathophysiology for POPF development. These differences are currently being researched and new explanations may lead to better POPF prevention management^47^.

The mortality rate after DP corresponded to the benchmark of the ISGPS Evidence Map of Pancreatic Surgery (0.8% *versus* less than 1% respectively), whereas the mortality rate of 2.1% after PD was higher than the 1% benchmark of the ISGPS Evidence Map of Pancreatic Surgery^5^. Major complications were observed more frequently in PD compared with DP (24.0% *versus* 18.7% respectively). These results raised the question as to why PD was associated with higher rates of mortality and complications, whereas DP had a higher risk of POPF. The leading reasons for POPFs being a clinical problem are that they cause haemorrhage due to vessel erosion or become infected^48^. Haemorrhages may be more frequent in PD because of the large area of resection and the location of central vessels in the surgical area causing higher complication and mortality rates. Clinically relevant infections of collections are more common after PD^48^. One reason for this may be infection with enterobacteria from the opened intestinal lumen or in the case of leakage from the hepatojejunostomy. In addition, drains are used more frequently during PD, which may result in ascending intra-abdominal infection. Furthermore, Nakamura *et al*.^49^ showed that a clinically relevant POPF is more likely to occur in PD due to microbial colonization of the bile in PD. Most fluid collections in DP resulting from POPFs are benign, do not lead to complications, and can be observed^50^. Finally, the lack of differentiation between POPF-related and non-POPF-related complications may be a reason for the higher complication rate after PD. PD is a considerably more complex operation, which has a higher risk of complications than DP.

Of further interest was the missing positive correlation between reoperations and re-interventions and major complications. This may lie in the lack of differentiation between POPF-related and non-POPF-related complications in most studies. Because a POPF is defined by its consequences, this distinction is important.

A meta-analysis of potential cost savings could not be performed because of the lack of information on treatment costs. Only Hassenpflug *et al*.^1^ conducted a full cost analysis. A clinically relevant POPF after DP was not significantly reduced by coverage with the teres ligament. However, there was a reduction in grade B/C POPFs of approximately 10%, which led to a significant reduction in the median total cost of treatment from €13 998 in the non-coverage group to €10 138 in the coverage group (*P* = 0.035)^1^. Other RCTs that only made projections of potential cost savings also reported cost reductions with additional coverage, even when artificial materials were used^26,28^. According to Maggino *et al*.^51^, hospital costs for grade B/C POPFs are 2.3 times higher than for an uncomplicated resection. With this in mind, cost savings play a major role in the debate regarding POPF prevention by coverage methods.

Comparing autologous and artificial coverage techniques, significant advantages regarding POPF reduction could only be demonstrated for autologous material. These results were in keeping with those of Tieftrunk *et al*.^7^, where autologous patch application proved superior in POPF prevention, compared with manual suture closure after DP. A similar result using sealants, such as TachoSil^®^ and fibrin glues, was not observed^7^. A benefit of autologous coverage could only be demonstrated in four studies^1,36–38^, of which the DISCOVER trial^1^ was weighted with almost 67.4%. Although the DISCOVER trial was unable to show significant POPF reduction, it was associated with less re-interventions and reoperations, as in the present meta-analysis. In the DISCOVER trial, the ligamentum teres hepatis was used, whereas the other three studies used the omentum and the seromuscular surface of the jejunum as coverage tissue. A network meta-analysis comparing eight different techniques and outcomes of remnant closure after DP claimed that coverage with the teres ligament was superior^11^. However, there remains a lack of autologous coverage studies, especially ones comparing the different techniques.

Whereas the present study was unable to show any benefit of artificial coverage in POPF prevention, the meta-analysis of Zhang *et al*.^9^ supported coverage with a polyglycolic acid mesh in PD and DP in their analysis of three RCTs and eight non-randomized trials^9^. Polyglycolic acid, one of many different types of artificial sealants, causes inflammation after being placed in the body, which in turn leads to infiltration of granulation tissue, and is absorbed within 2–3 months^29^. The multitude of different products for artificial sealing complicates the investigation of their benefits. In the present meta-analysis, 11 studies used fibrin-based sealants. Indeed, only Jang *et al*.^29^ and Bassi *et al*.^33^ analysed non-fibrin-based materials. Even when sealants were unable to significantly reduce the POPF rate, some slight effects, especially regarding the treatment of the left pancreatic remnant, were observed, as previously reported^52^. In addition, the present meta-analysis observed a reduction in the number of reoperations after artificial coverage. Carter *et al*.^39^ combined autologous and artificial coverage techniques by using a falciform patch with fibrin glue and compared it with conventional closure in DP. The POPF rates showed no significant difference, but some surrogate parameters revealed some advantages for the interventional group. However, it was the only study investigating a combination of techniques and further studies are required.

The present study has numerous limitations. First, one could question the informational value of pooled results garnered from different operations like PD and DP, combined with a variety of autologous and artificial coverage techniques. However, this problem was addressed by subgroup analyses. Second, some RCTs were small and lacked information regarding secondary outcomes. Third, significant variability regarding confounding factors, such as closure technique (for example handsewn *versus* stapler based), perioperative treatment, and different POPF and complication definitions within and between the included studies, needs to be considered when interpreting the results. However, this is also representative of the reality of clinical practice.

The determination of the best pancreatic remnant closure or anastomosis technique remains an important topic of research. The present meta-analysis showed the potential benefit of coverage techniques, especially those using autologous material, in POPF reduction and the mitigation of POPF postoperative burden. In addition, because no additional risk was observed using artificial or autologous coverage techniques, this approach can be recommended. It seemed that the benefit observed with coverage was higher in DP.

However, further RCTs are needed to delineate these advantages for each subgroup and to determine which techniques and materials should be used. To do so, RCTs should be performed with large populations, possibly in a multicentre setting, with clear stratification according to the mentioned POPF risk factors. Even if the best model for POPF prediction has not yet been fully decided, future studies should provide as much information as possible on all potential influencing factors. In addition, a combination of interventions should be avoided. Furthermore, surgical techniques need to be standardized and documented in detail in future RCTs. The same applies to patient follow-up treatment. Finally, each study should include all secondary endpoints mentioned in the present meta-analysis. It is important to assign these endpoints as either POPF-related or non-POPF-related complications. A total of eight RCTs are currently ongoing and yet to be published; there is a lack of information in the registry protocols about whether the criteria mentioned above have been met.

## Supplementary Material

zrae059_Supplementary_Data

## Data Availability

Processed data of the included RCTs are available from the Evidence Map of Pancreatic Surgery (www.evidencemap.surgery). Further data will be shared with other researchers on reasonable request.
