# Supplementary material for: Effect of artificial or autologous coverage of the pancreatic remnant or anastomosis on postoperative pancreatic fistulas after partial pancreatectomy: meta-analysis of randomized clinical trials
Source: BJS Open. 2024 May 30;8(3):zrae059. doi: 10.1093/bjsopen/zrae059 (PMC11138960; doi:10.1093/bjsopen/zrae059)
Supplement: zrae059_Supplementary_Data [file zrae059_supplementary_data.docx]

**Effect of Artificial or Autologous Coverage of the Pancreatic Remnant or Anastomosis on Postoperative Pancreatic Fistula after Partial Pancreatectomy: Meta-Analysis of Randomised Controlled Trials**

Walbe Walber Jonas K.^1^, Antony Pia^1^, Strothmann Hendrik^1^, Kalkum Eva^2^, Renzulli Pietro^1^, Hauswirth Fabian^1^, Probst Pascal^3^, Muller Markus K.^1,3^

**Affiliation**

^1^Department of Surgery, Cantonal Hospital Thurgau, Münsterlingen, Switzerland

^2^Study Centre of the German Society of Surgery (SDGC), University of Heidelberg, Heidelberg, Germany.

^3^Department of Surgery, Cantonal Hospital Thurgau, Frauenfeld, Switzerland

**Corresponding author**

Pascal Probst

Department of Surgery

Cantonal Hospital Thurgau

Pfaffenholzstrasse 4

CH-8501 Frauenfeld, Switzerland

T: +41 58 144 72 41

E-Mail: [pascal.probst@stgag.ch](mailto:matthias.pfister@usz.ch)

**Supplementary Materials - Index**

| **Supplementary Methods** |  |
| --- | --- |
| Search Strategy | *pag. 3* |
| Prisma Checklist  Prisma 2020 for Abstracts Checklist | *pag. 4-6*  *pag. 6* |
| **Supplementary Results** |  |
| Forest Plots of clinically relevant POPF rates (sensitivity analysis) | *pag. 7-9* |
| Forest Plots of mortality rates | *pag. 10-12* |
| Forest Plot of overall morbidity | *pag. 13* |
| Forest Plot of Clavien- Dindo >3 | *pag. 13* |
| Forest Plots of reoperation rates | *pag. 14-15* |
| Forest Plots of reintervention rates | *pag. 16-17* |
| Forest Plots of PPH rates | *pag. 17-18* |
| Forest Plots of rates of delayed gastric emptying | *pag. 19-20* |
| Forest Plots of rates of intra-abdominal collections | *pag. 20-21* |
| Forest Plots of SSI rates | *pag. 22-23* |
| Forest Plot of bile leak rates | *pag. 23* |
| Forest Plots of operation time | *pag. 24-25* |
| Forest Plots of intraoperative blood loss | *pag. 25-26* |
| Forest Plots of length of hospital stay | *pag. 27-28* |
|  |  |

**Supplementary Methods**

**Search Strategy**

Medline (via Pubmed):

((pancreas[MeSH terms] OR pancreas[tiab] OR pancreatic[tiab] OR pancreato*[tiab]) AND (resection* [tiab] OR surger* [tiab] OR surgical [tiab])) OR pancreaticoduodenectom*[tiab] OR pancreatoduodenectom*[tiab] OR Whipple*[tiab] OR ppWhipple*[tiab] OR PPPD[tiab] OR duodenopancreatectom*[tiab] OR pancreatectom*[tiab] OR “Pancreaticoduodenectomy”[Mesh] OR pancreatectomy[MeSH]

**AND** patch*[tiab] OR sealant*[tiab] OR coverag*[tiab] OR closure*[tiab] OR wrap*[tiab] OR fibrin[tiab] OR omental[tiab] OR "Anastomosis, Surgical/methods"[Mesh] OR "Anastomosis, Surgical/therapy"[Mesh] OR "Abdominal Wound Closure Techniques"[Mesh]

**AND** (randomised controlled trial [pt] OR "Controlled Clinical Trial" [pt] OR random*[tiab] OR RCT [tiab] OR "Randomised Controlled Trials as Topic"[Mesh])

**NOT** (animals [mh] NOT humans [mh])

CENTRAL:

((pancreas OR pancreatic OR pancreato*) NEAR/5 (resection* OR surger* OR surgical)):ti,ab,kw

OR (pancreaticoduodenectom* OR pancreatoduodenectom* OR Whipple* OR ppWhipple* OR PPPD OR duodenopancreatectom* OR pancreatectom*):ti,ab,kw

OR MeSH descriptor: [Pancreaticoduodenectomy] explode all trees OR [Pancreatectomy] explode all trees

**AND** (patch* OR sealant* OR coverag* OR closure* OR wrap* OR fibrin OR omental):ti,ab,kw

OR MeSH descriptor: [Anastomosis, Surgical] explode all trees and with qualifier(s): [methods - MT] OR [Anastomosis, Surgical] explode all trees and with qualifier(s): [therapy - TH] OR MeSH descriptor: [Abdominal Wound Closure Techniques] explode all trees

**Prisma checklist**

| **Section and Topic** | **Item #** | **Checklist item** | **Location where item is reported** |
| --- | --- | --- | --- |
| **TITLE** | | |  |
| Title | 1 | Identify the report as a systematic review. | 1 |
| **ABSTRACT** | | |  |
| Abstract | 2 | See the PRISMA 2020 for Abstracts checklist. | Suppl. 6 |
| **INTRODUCTION** | | |  |
| Rationale | 3 | Describe the rationale for the review in the context of existing knowledge. | 3 |
| Objectives | 4 | Provide an explicit statement of the objective(s) or question(s) the review addresses. | 3 |
| **METHODS** | | |  |
| Eligibility criteria | 5 | Specify the inclusion and exclusion criteria for the review and how studies were grouped for the syntheses. | 4 |
| Information sources | 6 | Specify all databases, registers, websites, organisations, reference lists and other sources searched or consulted to identify studies. Specify the date when each source was last searched or consulted. | 4 |
| Search strategy | 7 | Present the full search strategies for all databases, registers and websites, including any filters and limits used. | Suppl. 3 |
| Selection process | 8 | Specify the methods used to decide whether a study met the inclusion criteria of the review, including how many reviewers screened each record and each report retrieved, whether they worked independently, and if applicable, details of automation tools used in the process. | 5 |
| Data collection process | 9 | Specify the methods used to collect data from reports, including how many reviewers collected data from each report, whether they worked independently, any processes for obtaining or confirming data from study investigators, and if applicable, details of automation tools used in the process. | 5 |
| Data items | 10a | List and define all outcomes for which data were sought. Specify whether all results that were compatible with each outcome domain in each study were sought (e.g. for all measures, time points, analyses), and if not, the methods used to decide which results to collect. | 5 |
|  | 10b | List and define all other variables for which data were sought (e.g. participant and intervention characteristics, funding sources). Describe any assumptions made about any missing or unclear information. | 7  Table 1 |
| Study risk of bias assessment | 11 | Specify the methods used to assess risk of bias in the included studies, including details of the tool(s) used, how many reviewers assessed each study and whether they worked independently, and if applicable, details of automation tools used in the process. | 5 |
| Effect measures | 12 | Specify for each outcome the effect measure(s) (e.g. risk ratio, mean difference) used in the synthesis or presentation of results. | 5 |
| Synthesis methods | 13a | Describe the processes used to decide which studies were eligible for each synthesis (e.g. tabulating the study intervention characteristics and comparing against the planned groups for each synthesis (item #5)). | 4, 8 |
|  | 13b | Describe any methods required to prepare the data for presentation or synthesis, such as handling of missing summary statistics, or data conversions. | 5,6,8 |
|  | 13c | Describe any methods used to tabulate or visually display results of individual studies and syntheses. | 5 |
|  | 13d | Describe any methods used to synthesize results and provide a rationale for the choice(s). If meta-analysis was performed, describe the model(s), method(s) to identify the presence and extent of statistical heterogeneity, and software package(s) used. | 5,6 |
|  | 13e | Describe any methods used to explore possible causes of heterogeneity among study results (e.g. subgroup analysis, meta-regression). | 5,6 |
|  | 13f | Describe any sensitivity analyses conducted to assess robustness of the synthesized results. | 8,9 |
| Reporting bias assessment | 14 | Describe any methods used to assess risk of bias due to missing results in a synthesis (arising from reporting biases). | 5 |
| Certainty assessment | 15 | Describe any methods used to assess certainty (or confidence) in the body of evidence for an outcome. | 5 |
| **RESULTS** | | |  |
| Study selection | 16a | Describe the results of the search and selection process, from the number of records identified in the search to the number of studies included in the review, ideally using a flow diagram. | 7  Figure 1 |
|  | 16b | Cite studies that might appear to meet the inclusion criteria, but which were excluded, and explain why they were excluded. | 7  Figure 1 |
| Study characteristics | 17 | Cite each included study and present its characteristics. | 7 |
| Risk of bias in studies | 18 | Present assessments of risk of bias for each included study. | Figure 2 |
| Results of individual studies | 19 | For all outcomes, present, for each study: (a) summary statistics for each group (where appropriate) and (b) an effect estimate and its precision (e.g. confidence/credible interval), ideally using structured tables or plots. | Suppl. 7-28  Figure 3 |
| Results of syntheses | 20a | For each synthesis, briefly summarise the characteristics and risk of bias among contributing studies. | 7  Table 1  Figure 2 |
|  | 20b | Present results of all statistical syntheses conducted. If meta-analysis was done, present for each the summary estimate and its precision (e.g. confidence/credible interval) and measures of statistical heterogeneity. If comparing groups, describe the direction of the effect. | 8-10  Suppl. 7-28 |
|  | 20c | Present results of all investigations of possible causes of heterogeneity among study results. | 8  Suppl. 7-28  Figure 3 |
|  | 20d | Present results of all sensitivity analyses conducted to assess the robustness of the synthesized results. | 8,9 |
| Reporting biases | 21 | Present assessments of risk of bias due to missing results (arising from reporting biases) for each synthesis assessed. | Figure 2 |
| Certainty of evidence | 22 | Present assessments of certainty (or confidence) in the body of evidence for each outcome assessed. | 8-10  Suppl. 7-28  Figure 3 |
| **DISCUSSION** | | |  |
| Discussion | 23a | Provide a general interpretation of the results in the context of other evidence. | 11-13 |
|  | 23b | Discuss any limitations of the evidence included in the review. | 13 |
|  | 23c | Discuss any limitations of the review processes used. | 13 |
|  | 23d | Discuss implications of the results for practice, policy, and future research. | 11-14 |
| **OTHER INFORMATION** | | |  |
| Registration and protocol | 24a | Provide registration information for the review, including register name and registration number, or state that the review was not registered. | 14 |
|  | 24b | Indicate where the review protocol can be accessed, or state that a protocol was not prepared. | 14 |
|  | 24c | Describe and explain any amendments to information provided at registration or in the protocol. | 14 |
| Support | 25 | Describe sources of financial or non-financial support for the review, and the role of the funders or sponsors in the review. | 1 |
| Competing interests | 26 | Declare any competing interests of review authors. | 14 |
| Availability of data, code and other materials | 27 | Report which of the following are publicly available and where they can be found: template data collection forms; data extracted from included studies; data used for all analyses; analytic code; any other materials used in the review. | 10,12,15-17  Table 2 |

**Prisma 2020 for Abstracts Checklist**

| **Section and Topic** | **Item #** | **Checklist item** | **Reported (Yes/No)** |
| --- | --- | --- | --- |
| **TITLE** | | |  |
| Title | 1 | Identify the report as a systematic review. | Yes |
| **BACKGROUND** | | |  |
| Objectives | 2 | Provide an explicit statement of the main objective(s) or question(s) the review addresses. | Yes |
| **METHODS** | | |  |
| Eligibility criteria | 3 | Specify the inclusion and exclusion criteria for the review. | Yes |
| Information sources | 4 | Specify the information sources (e.g. databases, registers) used to identify studies and the date when each was last searched. | Yes |
| Risk of bias | 5 | Specify the methods used to assess risk of bias in the included studies. | No |
| Synthesis of results | 6 | Specify the methods used to present and synthesise results. | No |
| **RESULTS** | | |  |
| Included studies | 7 | Give the total number of included studies and participants and summarise relevant characteristics of studies. | Yes |
| Synthesis of results | 8 | Present results for main outcomes, preferably indicating the number of included studies and participants for each. If meta-analysis was done, report the summary estimate and confidence/credible interval. If comparing groups, indicate the direction of the effect (i.e. which group is favoured). | Yes |
| **DISCUSSION** | | |  |
| Limitations of evidence | 9 | Provide a brief summary of the limitations of the evidence included in the review (e.g. study risk of bias, inconsistency and imprecision). | Yes |
| Interpretation | 10 | Provide a general interpretation of the results and important implications. | Yes |
| **OTHER** | | |  |
| Funding | 11 | Specify the primary source of funding for the review. | Yes |
| Registration | 12 | Provide the register name and registration number. | Yes |

**Supplementary Results**

| **Figure 1 Forest Plot of all studies for the clinically relevant POPF rates (sensitivity analysis) according to type of coverage** |
| --- |
| 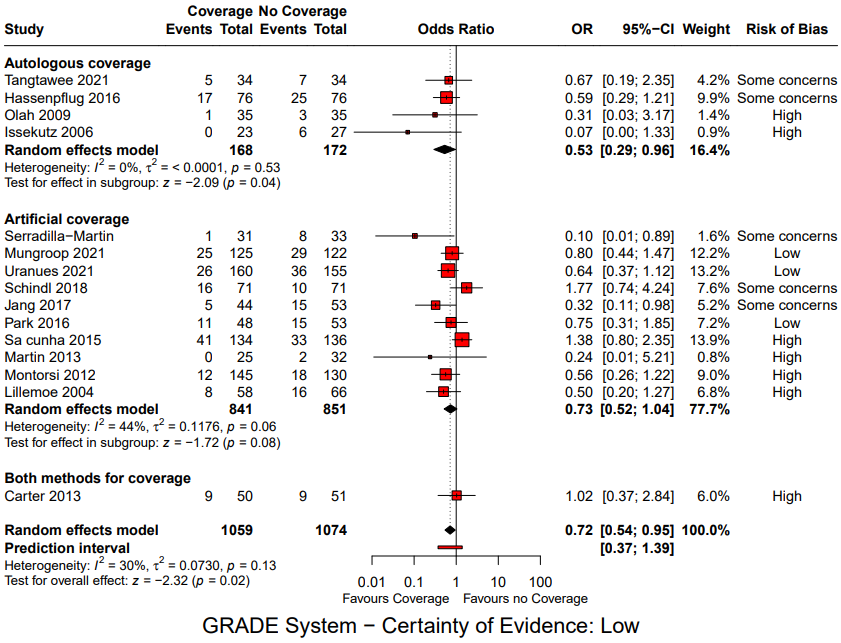 |

**Reasons for downgrading:** RoB -1 level, Indirectness 1 level

| **Figure 2 Forest Plot of all studies for the clinically relevant POPF rates (sensitivity analysis) according to type of operation** |
| --- |
| 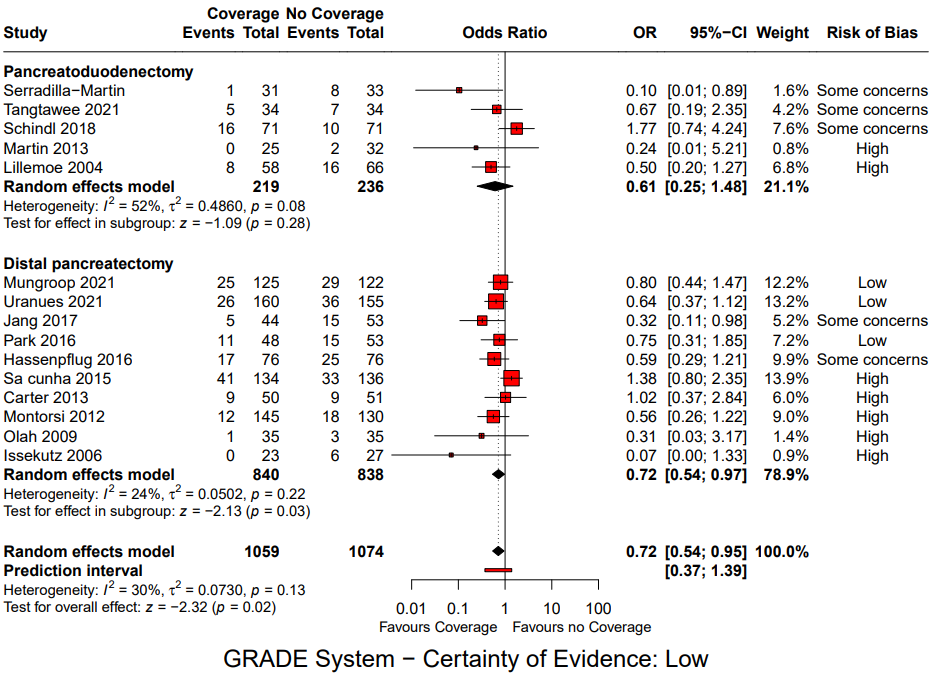 |

**Reasons for downgrading:** RoB -1 level, Indirectness 1 level

| **Figure 3 Forest Plot of studies with artificial coverage and clinically relevant POPF rates (sensitivity analysis)** |
| --- |
| 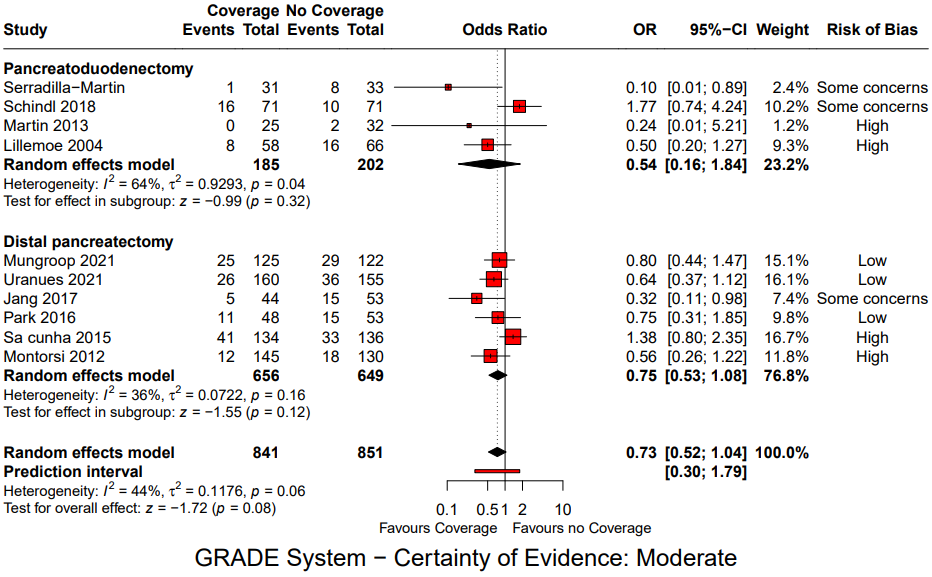 |

**Reasons for downgrading:** RoB -1 level

| **Figure 4 Forest Plot of studies with autologous coverage and clinically relevant POPF rates (sensitivity analysis)** |
| --- |
| 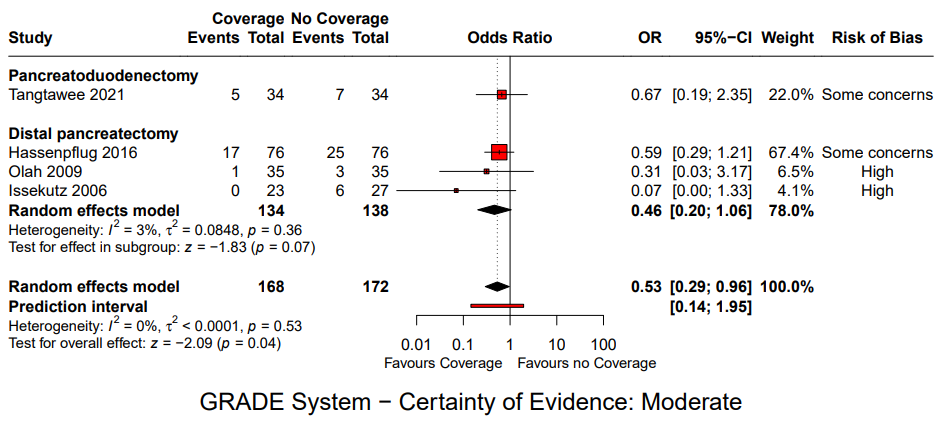 |

**Reasons for downgrading:** RoB -1 level

| **Figure 5 Forest Plot of all studies for mortality rates according to type of coverage** |
| --- |
| 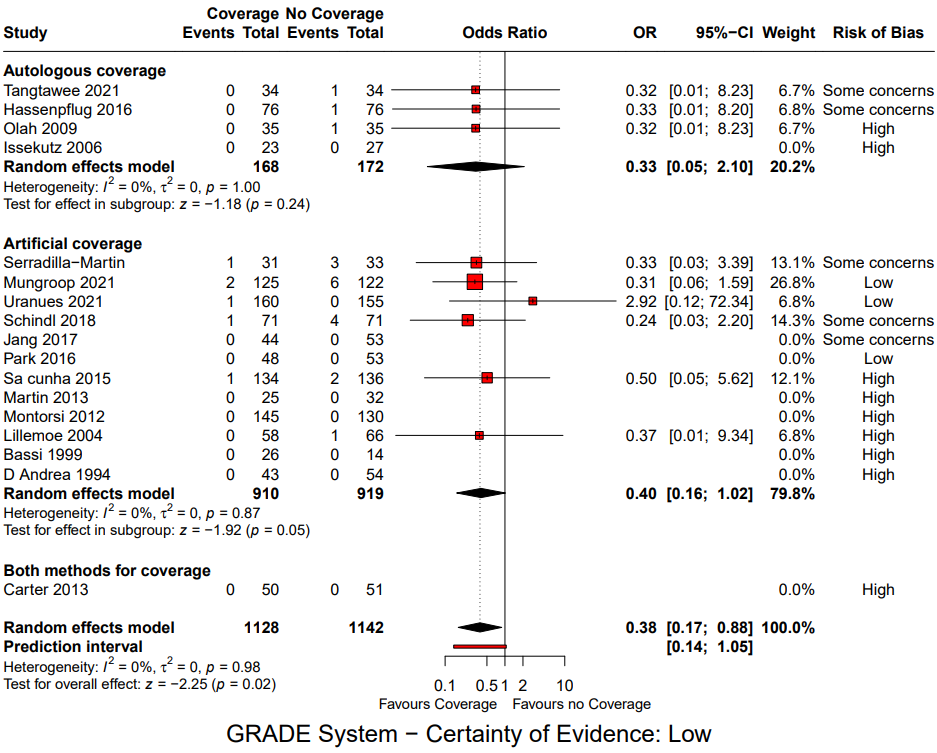 |

**Reasons for downgrading:** RoB -2 levels

| **Figure 6 Forest Plot of all studies for mortality rates according to type of operation** |
| --- |
| 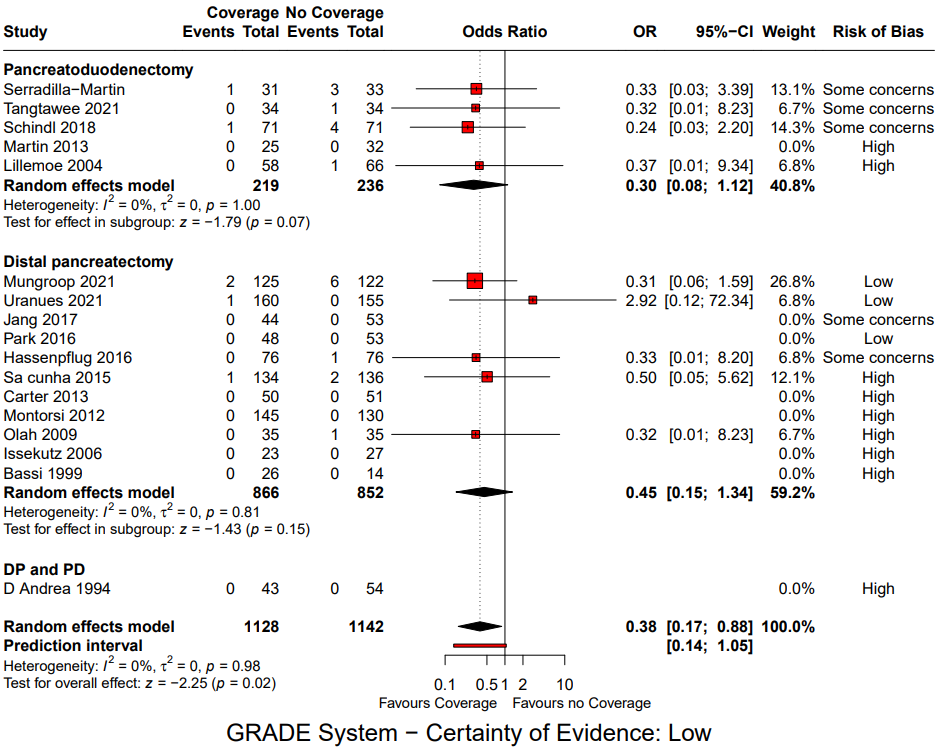 |

**Reasons for downgrading:** RoB -2 levels

| **Figure 7 Forest Plot of studies with artificial coverage and mortality rates** |
| --- |
| 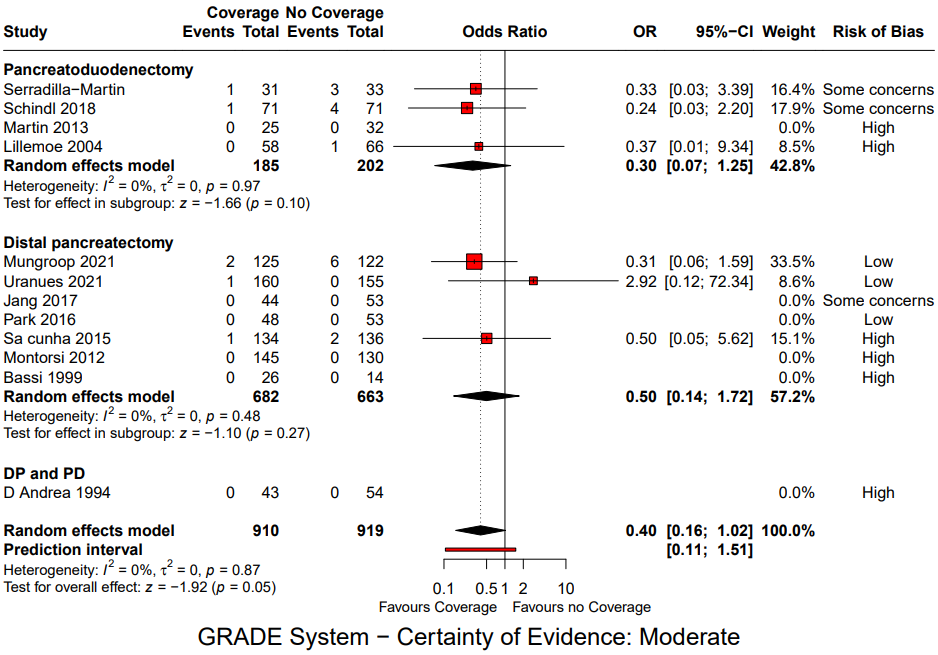 |

**Reasons for downgrading:** RoB -1 level

| **Figure 8 Forest Plot of studies with autologous coverage and mortality rates** |
| --- |
| 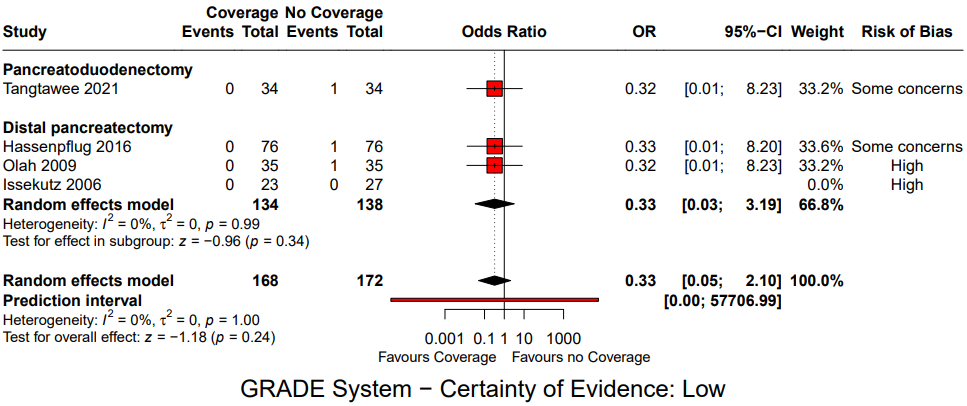 |

**Reasons for downgrading:** RoB -1 level, Imprecision -1 level

| **Figure 9 Forest Plot of all studies for overall morbidity according to type of operation (studies with autologous coverage did not exist for this category)** |
| --- |
| 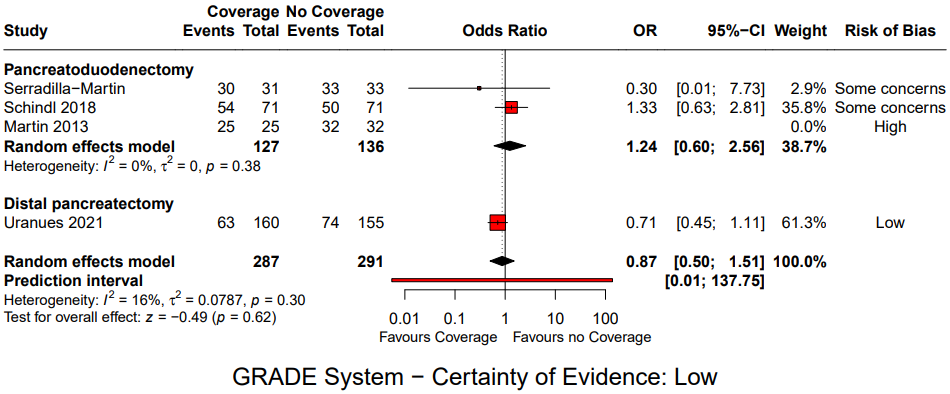 |

**Reasons for downgrading:** RoB -1 level, Imprecision -1 level

| **Figure 10 Forest Plot of all studies for Clavien- Dindo >3 according to type of operation (studies with autologous coverage did not exist for this category)** |
| --- |
| 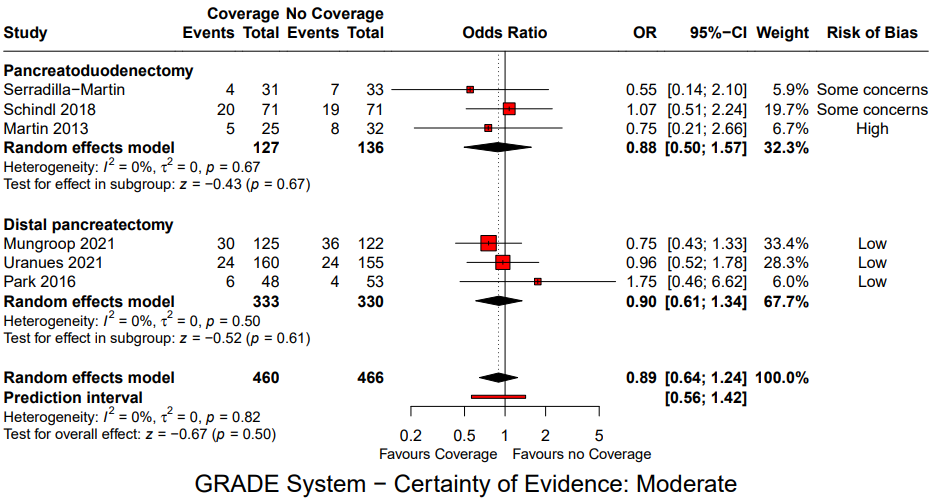 |

**Reasons for downgrading:** RoB -1 level

| **Figure 11 Forest Plot of all studies for reoperation rates according to type of coverage** |
| --- |
| 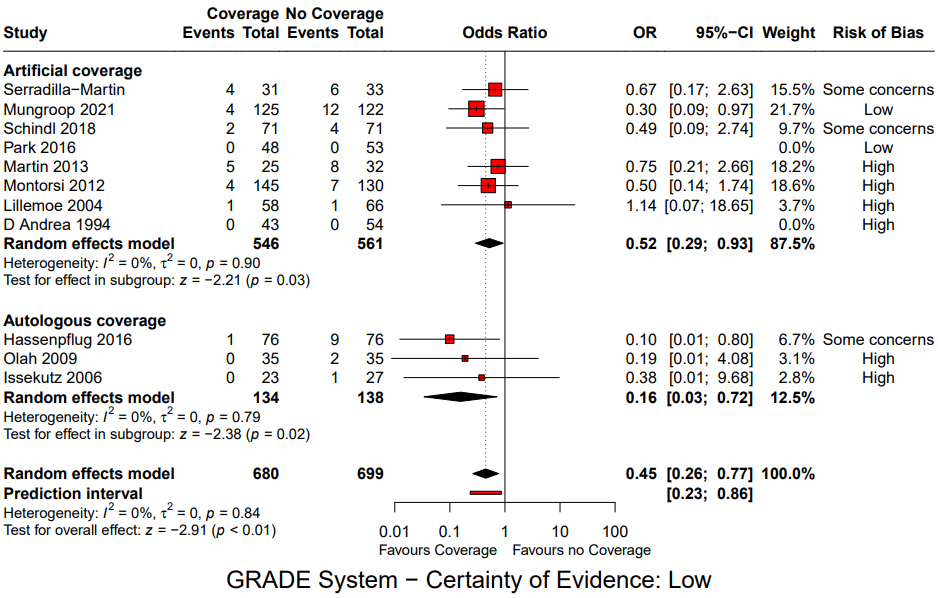 |

**Reasons for downgrading:** RoB -2 levels

| **Figure 12 Forest Plot of all studies for reoperation rates according to type of operation** |
| --- |
| 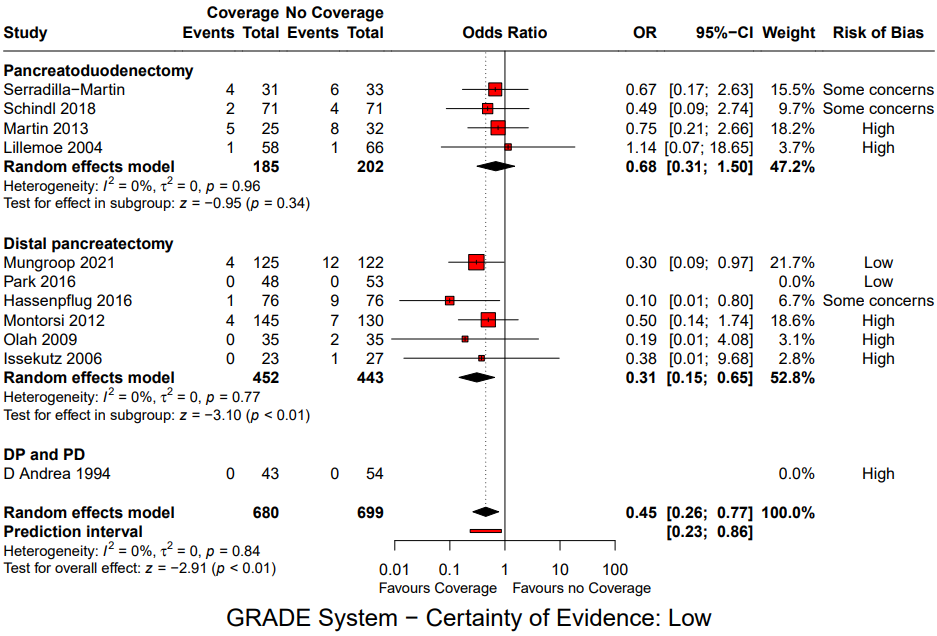 |

**Reasons for downgrading:** RoB -2 levels

| **Figure 13 Forest Plot of studies with artificial coverage and reoperation rates** |
| --- |
| 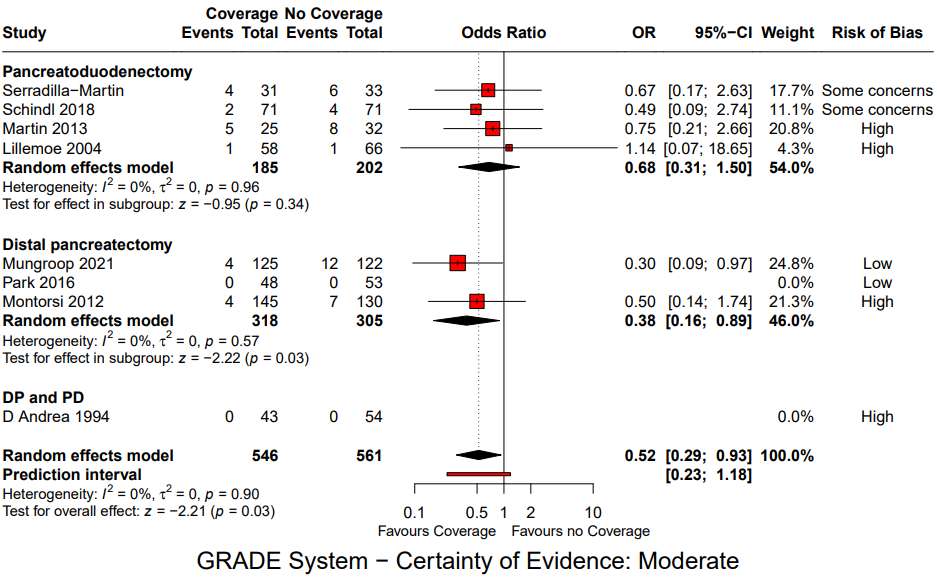 |

**Reasons for downgrading:** RoB -1 level

**Figure 14 Forest Plot of studies with autologous coverage after DP and reoperation rates**


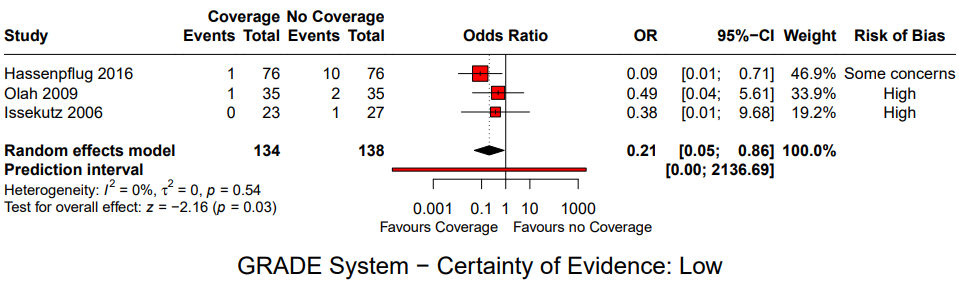


| **Reasons for downgrading:** RoB -2 levels  **Figure 15 Forest Plot of all studies for reintervention rates according to type of coverage** |
| --- |
| 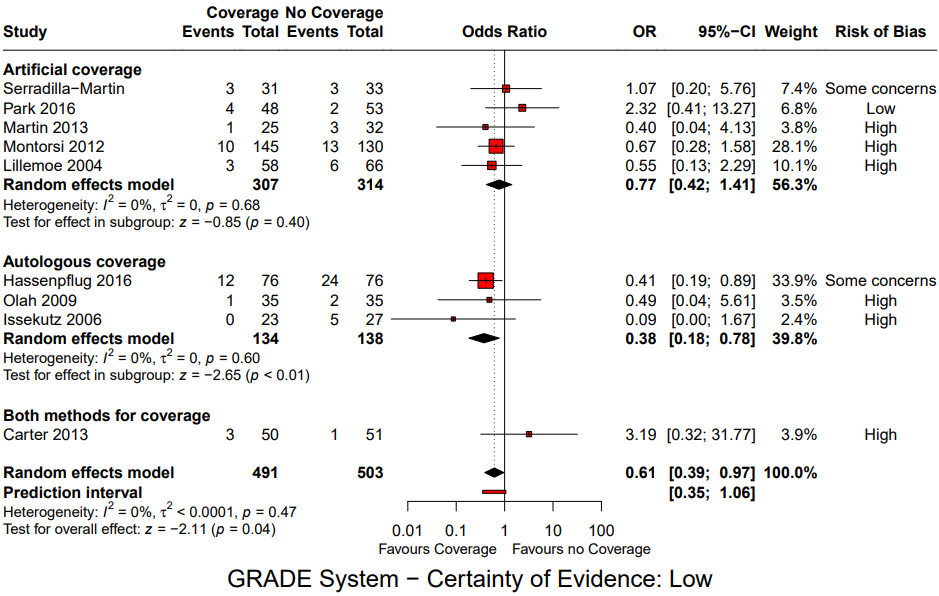 |

**Reasons for downgrading:** RoB -2 levels

| **Figure 16 Forest Plot of all studies for reintervention rates according to type of operation** |
| --- |
| 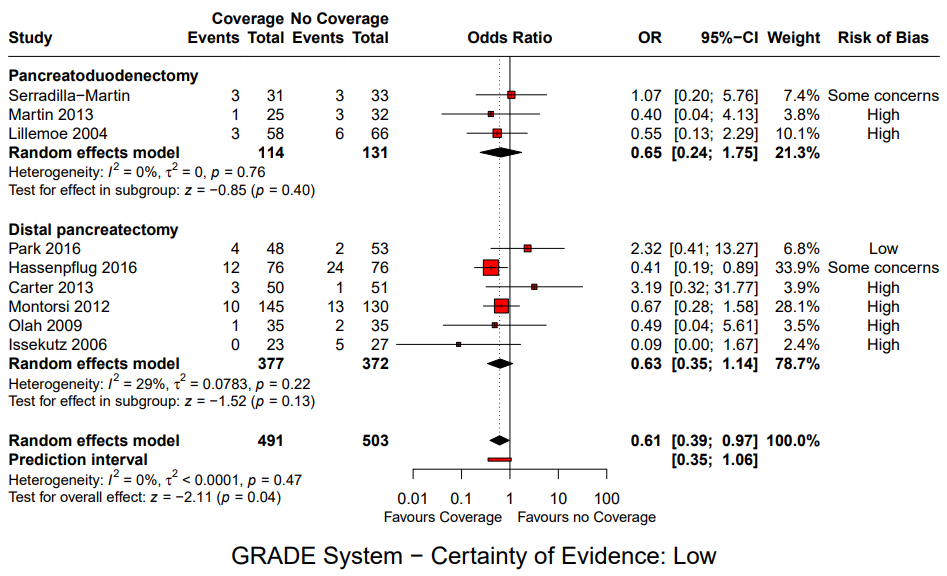 |

**Reasons for downgrading:** RoB -2 levels

| **Figure 17 Forest Plot of studies with artificial coverage and reintervention rates** |
| --- |
| 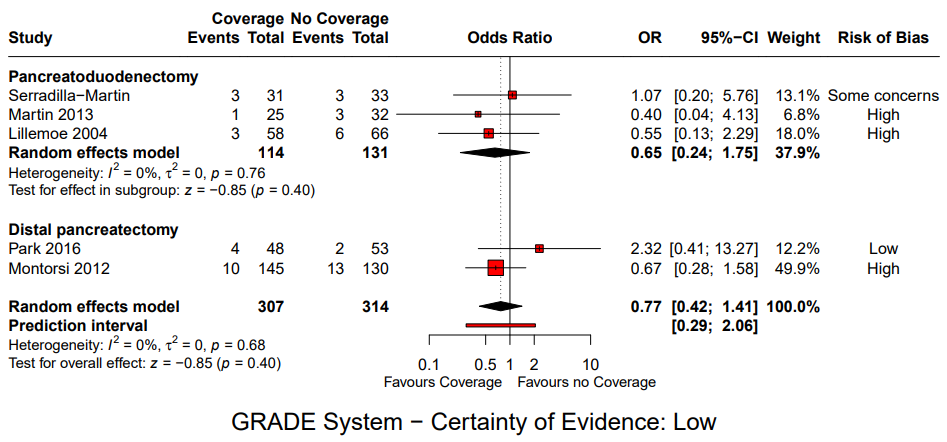 |

**Reasons for downgrading:** RoB -2 levels

**Figure 18 Forest Plot of studies with autologous coverage after DP and reintervention rates**

**
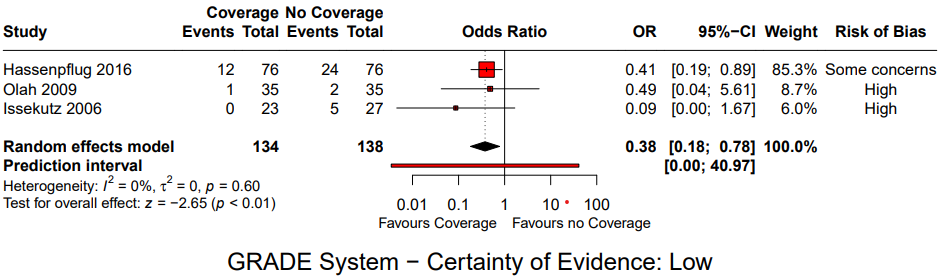
**

**Reasons for downgrading:** RoB -2 levels

| **Figure 19 Forest Plot of all studies for PPH rates according to type of coverage** |
| --- |
| 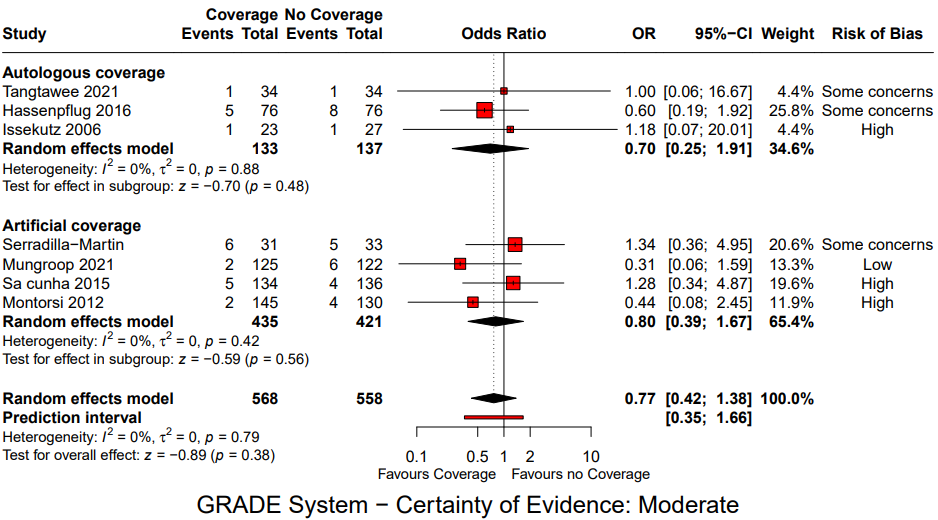 |

**Reasons for downgrading:** RoB -1 level

| **Figure 20 Forest Plot of all studies for PPH rates according to type of operation** |
| --- |
| 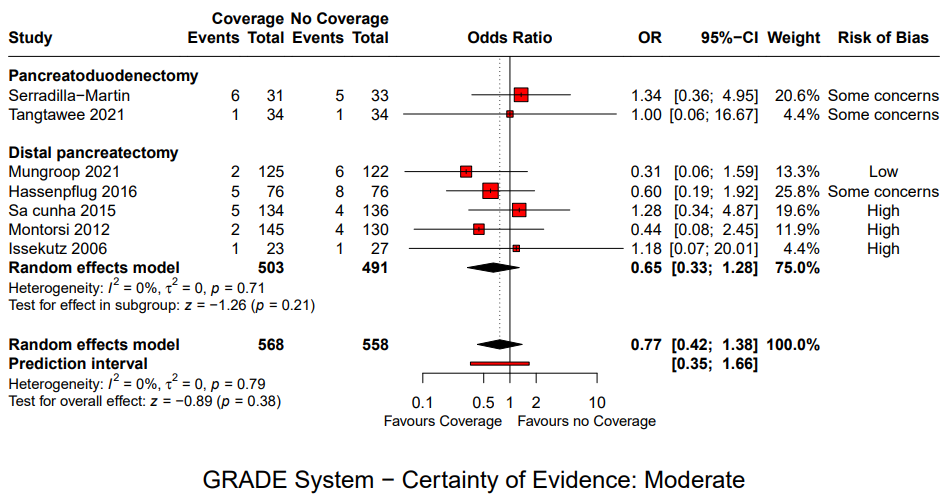 |

**Reasons for downgrading:** RoB -1 level

| **Figure 21 Forest Plot of studies with artificial coverage and PPH rates** |
| --- |
| 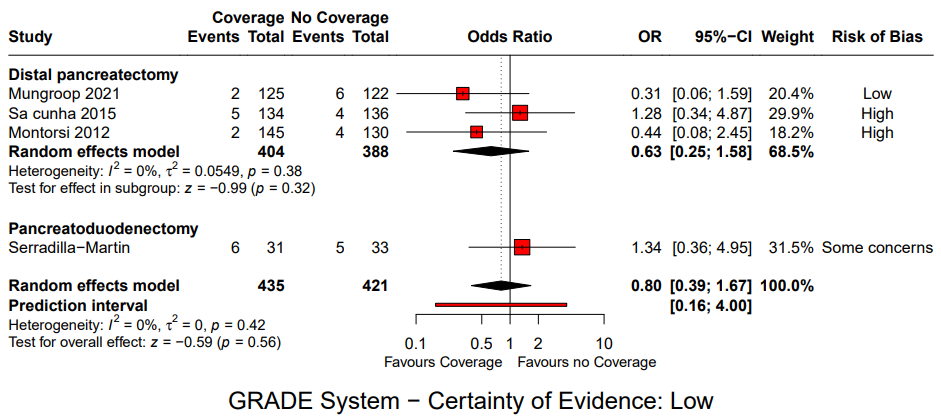 |

**Reasons for downgrading:** RoB -1 level, Imprecision -1 level

| **Figure 22 Forest Plot of studies with autologous coverage and PPH rates**  **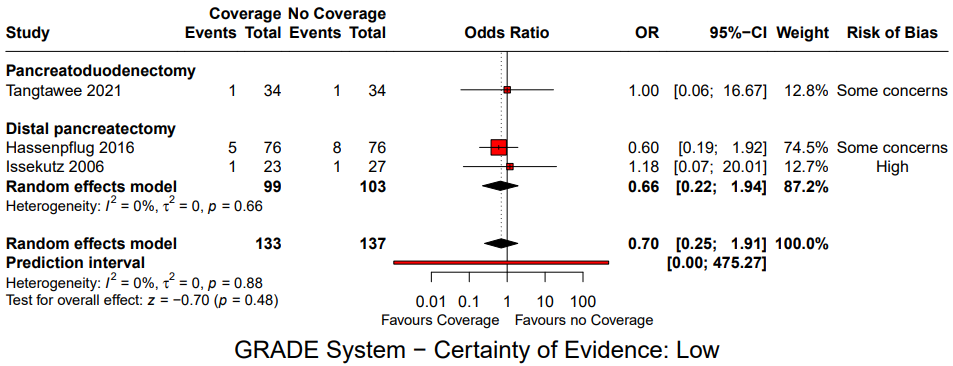**  **Reasons for downgrading:** RoB -1 level, Imprecision -1 level  **Figure 23 Forest Plot of all studies for rates of delayed gastric emptying according to type of coverage** |
| --- |
| 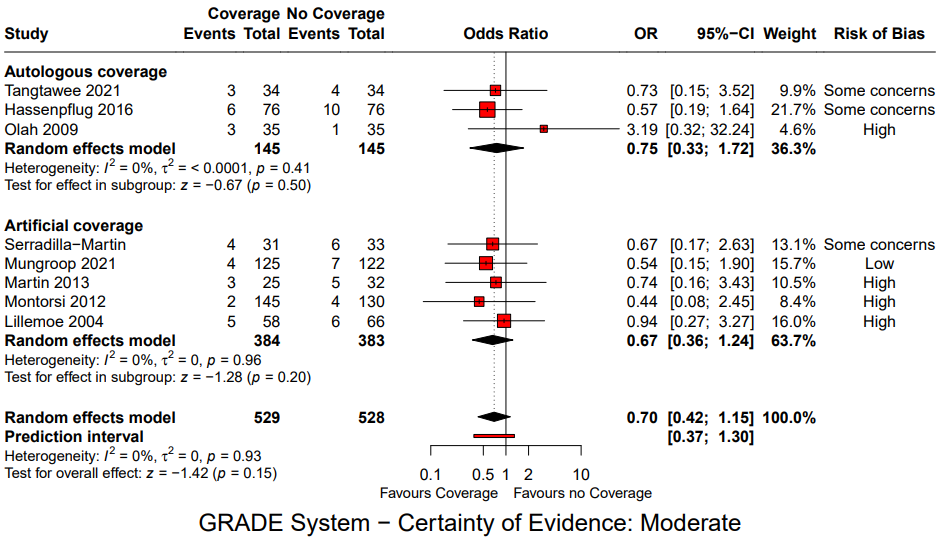 |

**Reasons for downgrading:** RoB -1 level

| **Figure 24 Forest Plot of all studies for rates of delayed gastric emptying according to type of operation** |
| --- |
| 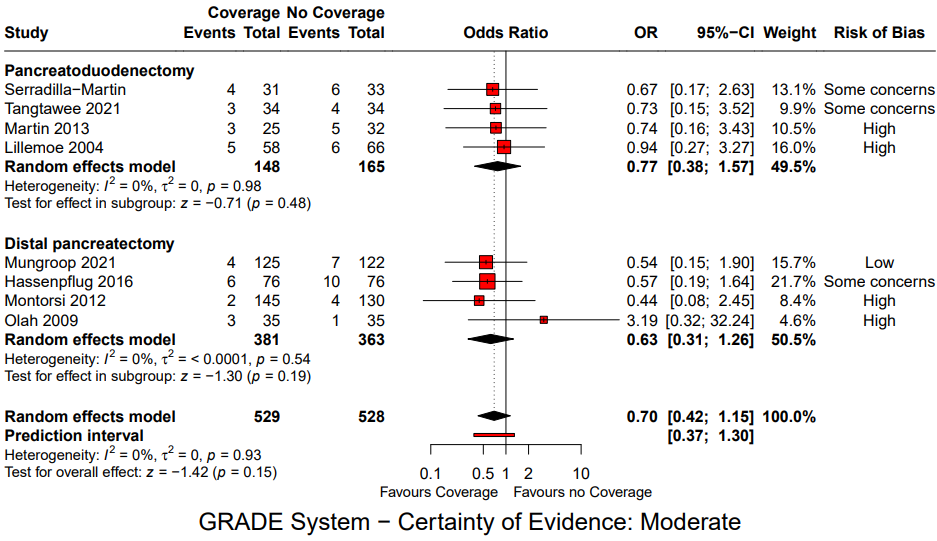 |

**Reasons for downgrading:** RoB -1 level

| **Figure 25 Forest Plot of studies with artificial coverage and rates of delayed gastric emptying** |
| --- |
| 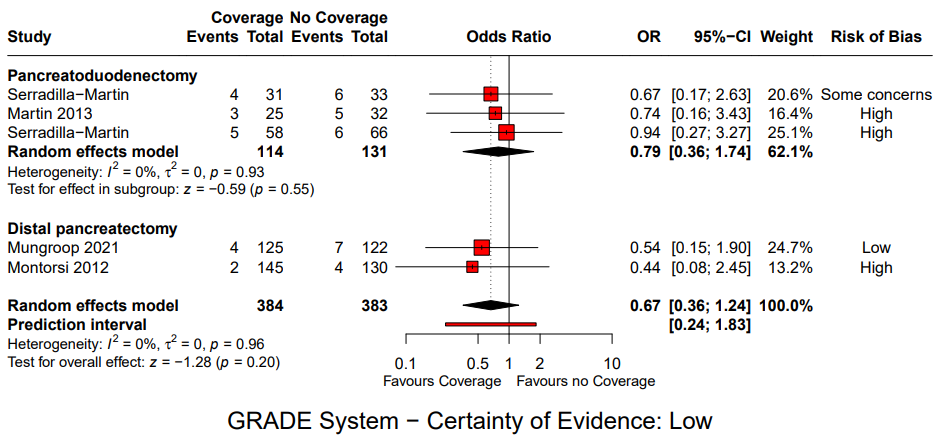 |

**Reasons for downgrading:** RoB -2 levels

**Figure 26 Forest Plot of studies with autologous coverage and rates of delayed gastric emptying**


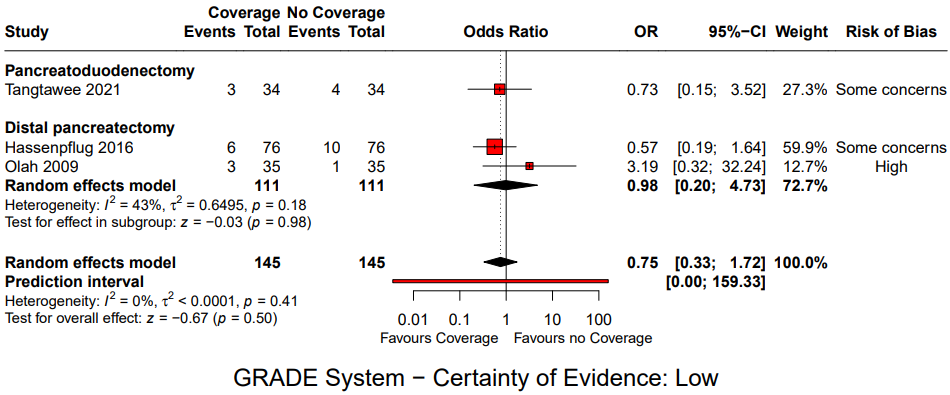


**Reasons for downgrading:** RoB -1 level, Imprecision -1 level

| **Figure 27 Forest Plot of all studies for rates of intra-abdominal collections according to type of coverage** |
| --- |
| 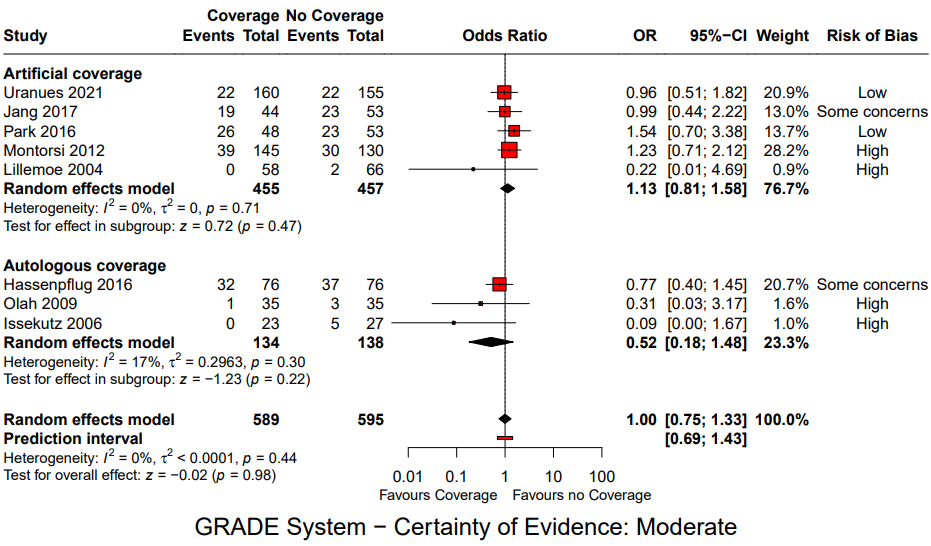 |

**Reasons for downgrading:** RoB -1 level

| **Figure 28 Forest Plot of all studies for rates of intra-abdominal collections according to type of operation** |
| --- |
| 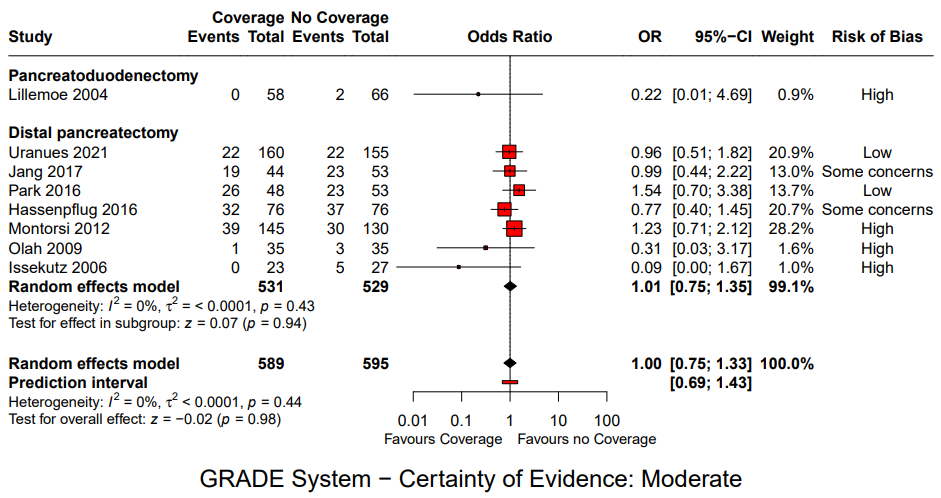 |

**Reasons for downgrading:** RoB -1 level

| **Figure 29 Forest Plot of studies with artificial coverage and rates of intra-abdominal collections** |
| --- |
| 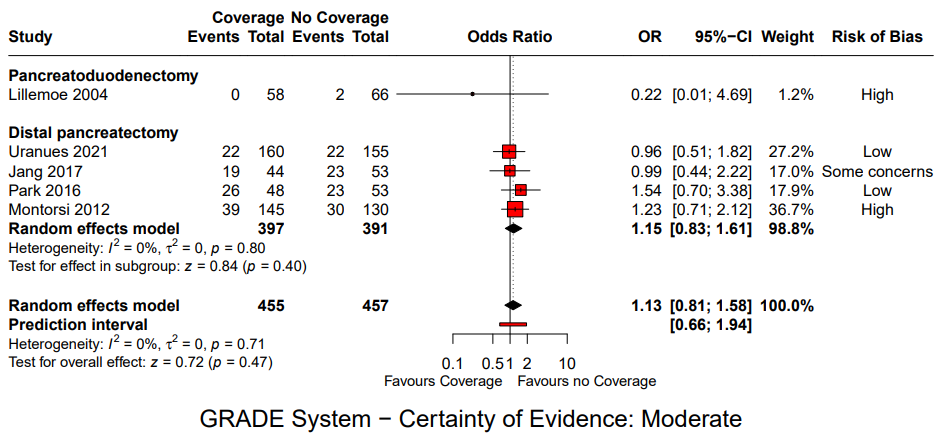 |

**Reasons for downgrading:** RoB -1 level

| **Figure 30 Forest Plot of studies with autologous coverage after DP and rates of intra-abdominal collections**  **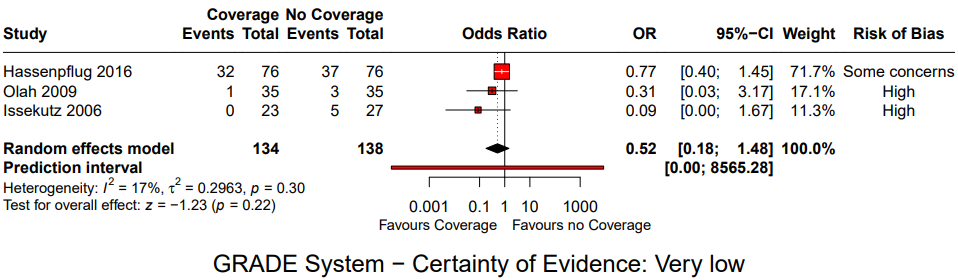**  **Reasons for downgrading:** RoB -2 levels, Imprecision -1 level  **Figure 31 Forest Plot of all studies for SSI rates according to type of coverage** |
| --- |
| 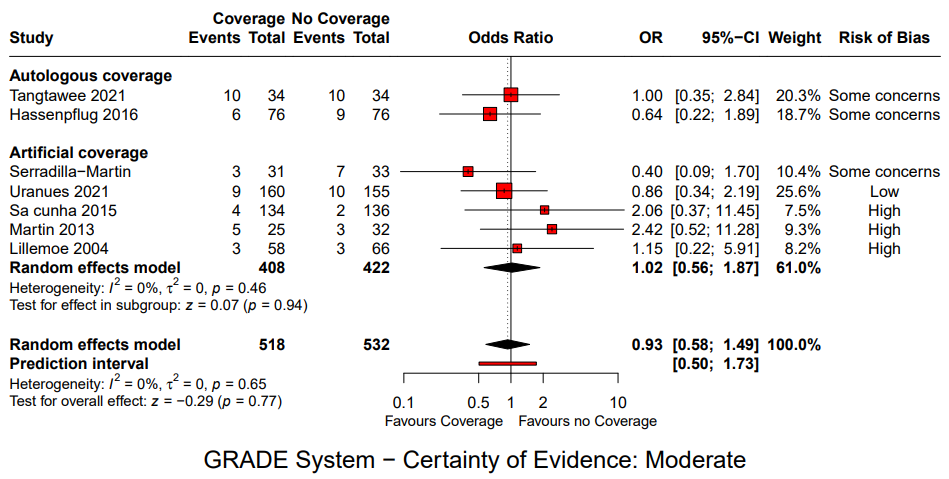 |

**Reasons for downgrading:** RoB -1 level

| **Figure 32 Forest Plot of all studies for SSI rates according to type of operation** |
| --- |
| 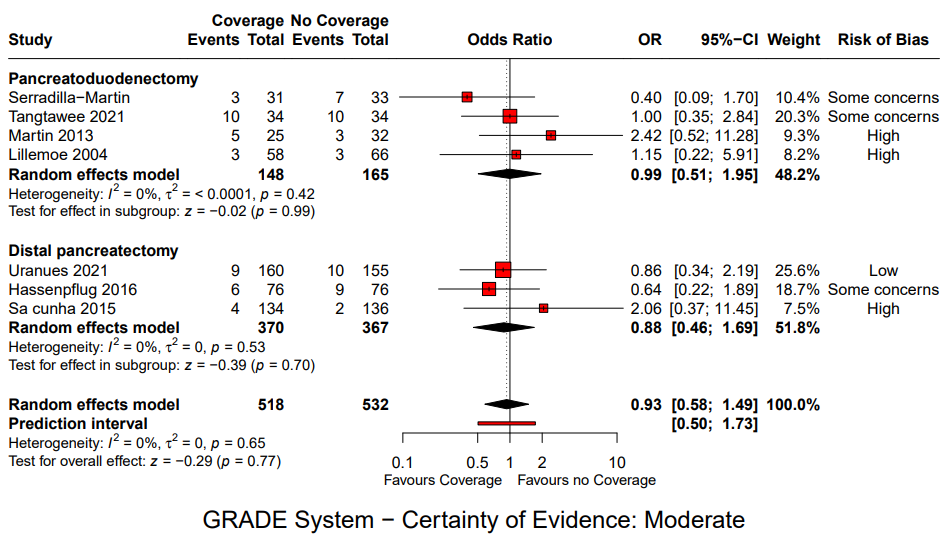 |

**Reasons for downgrading:** RoB -1 level

| **Figure 33 Forest Plot of studies with artificial coverage and SSI rates** |
| --- |
| **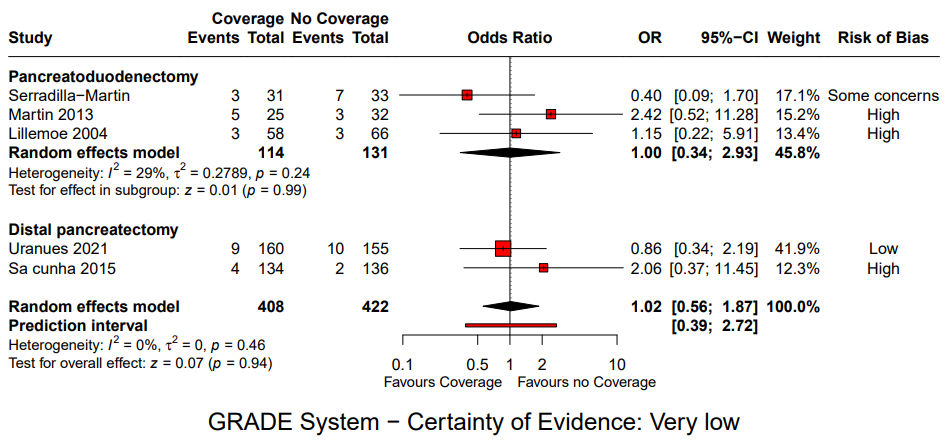** |

**Reasons for downgrading:** RoB -2 levels, Imprecision -1 level

| **Figure 34 Forest Plot of all studies for bile leak rates according to type of operation (studies with autologous coverage did not exist for this category)** |
| --- |
| 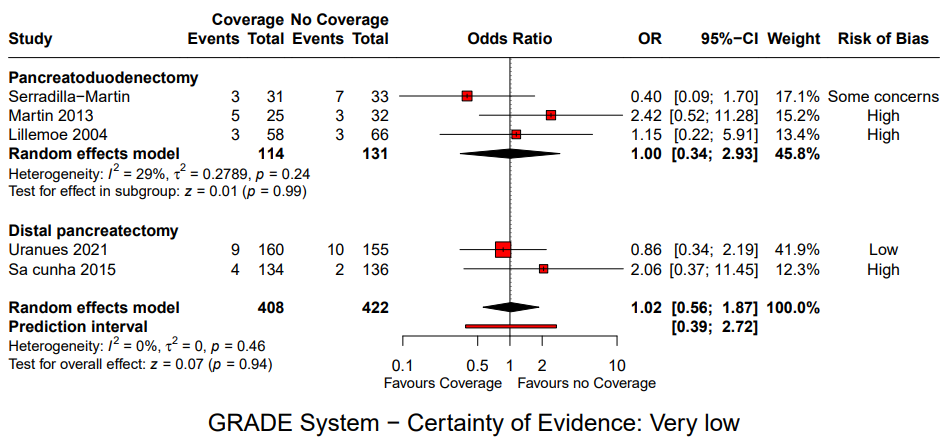 |

**Reasons for downgrading:** RoB -2 levels, Imprecision -1 level

| **Figure 35 Forest Plot of all studies for operation time according to type of coverage** |
| --- |
| 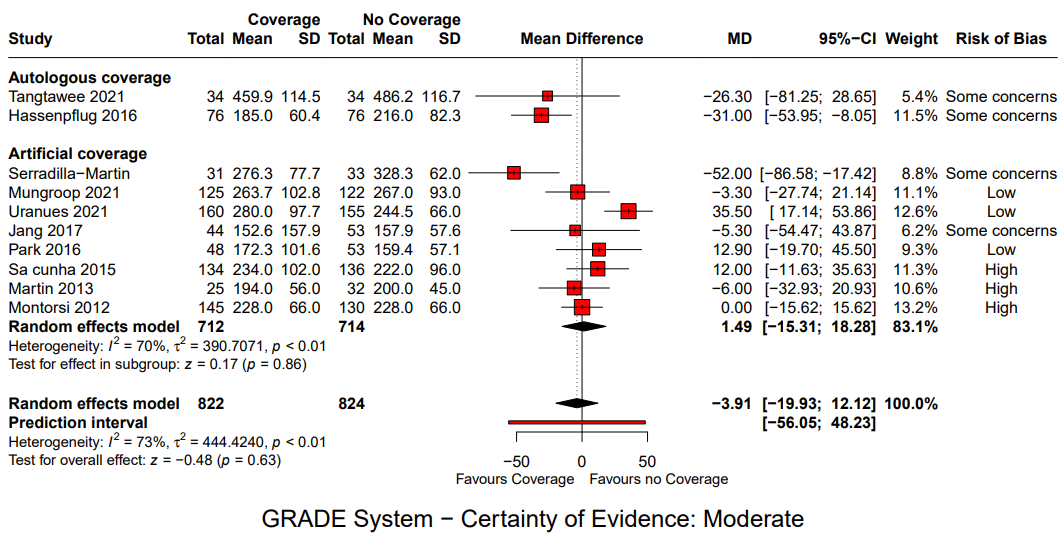 |

**Reasons for downgrading:** RoB -1 level

| **Figure 36 Forest Plot of all studies for operation time according to type of operation** |
| --- |
| 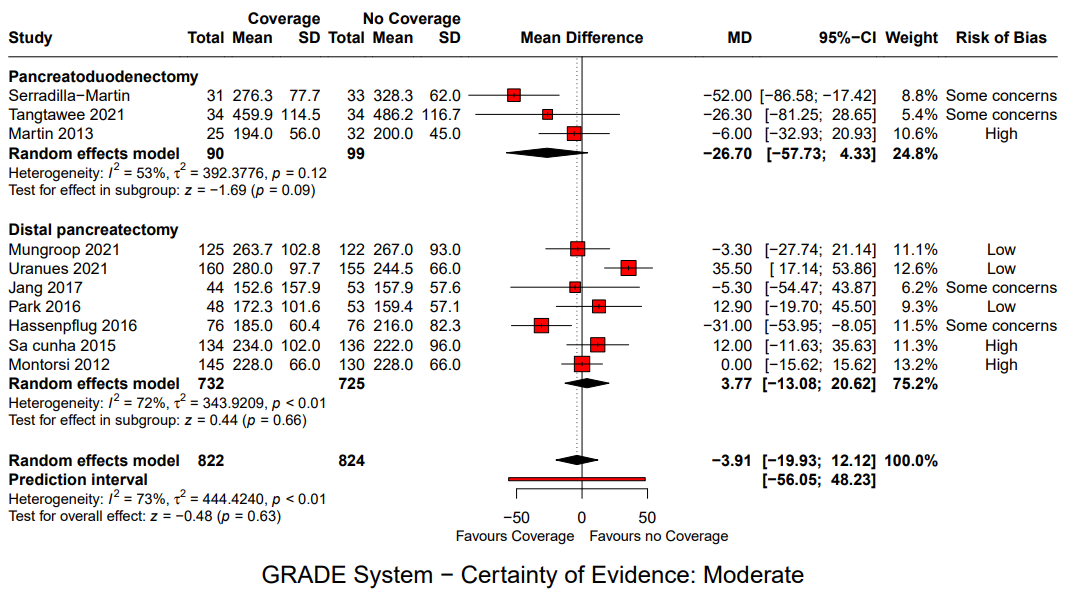 |

**Reasons for downgrading:** RoB -1 level

| **Figure 37 Forest Plot of studies with artificial coverage and operation time** |
| --- |
| 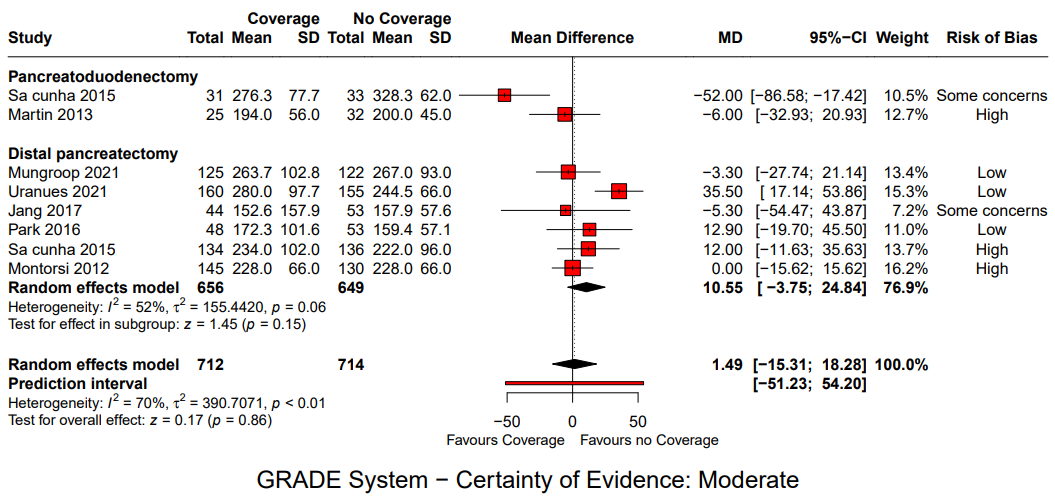 |

**Reasons for downgrading:** RoB -1 level

| **Figure 38 Forest Plot of all studies for intraoperative blood loss according to type of coverage** |
| --- |
| **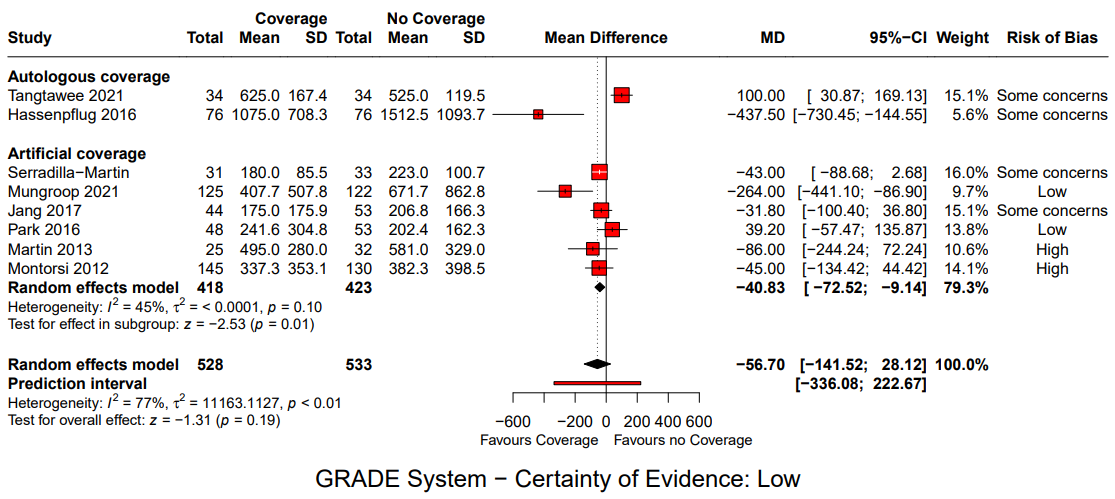** |

**Reasons for downgrading:** RoB -1 level, Inconsistency -1 level

| **Figure 39 Forest Plot of all studies for intraoperative blood loss according to type of operation** |
| --- |
| **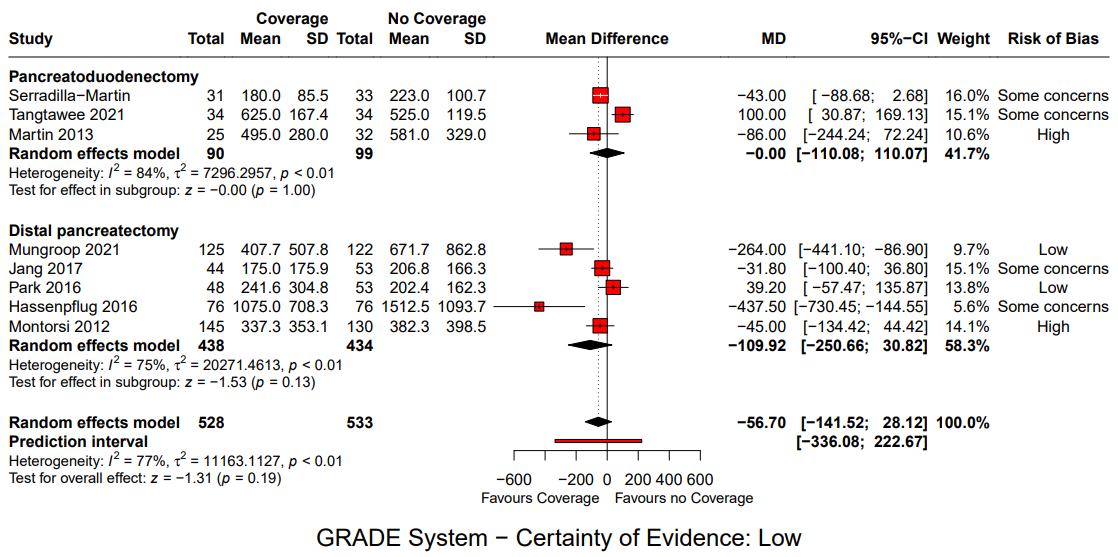** |

**Reasons for downgrading:** RoB -1 level, Inconsistency -1 level

| **Figure 40 Forest Plot of studies with artificial coverage and intraoperative blood loss** |
| --- |
| **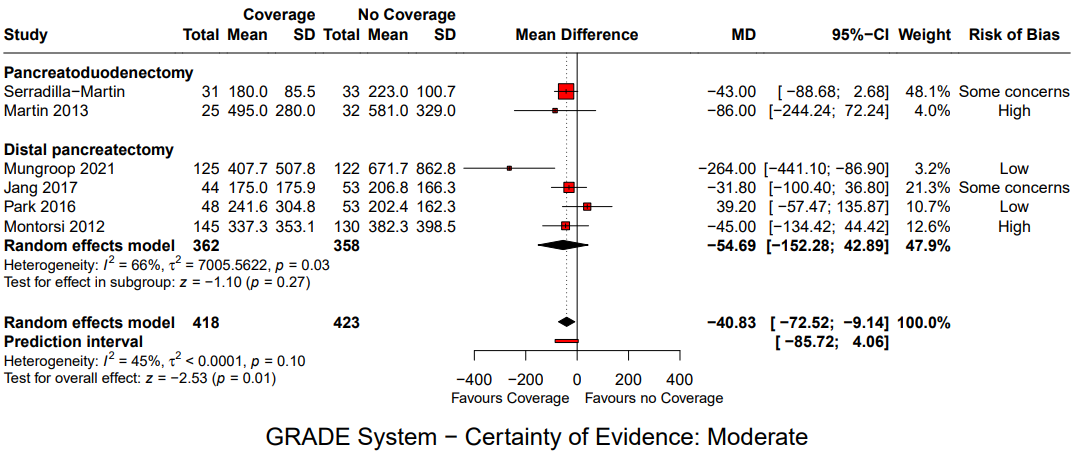** |

**Reasons for downgrading:** RoB -1 level

| **Figure 41 Forest Plot of all studies for length of hospital stay according to type of coverage** |
| --- |
| **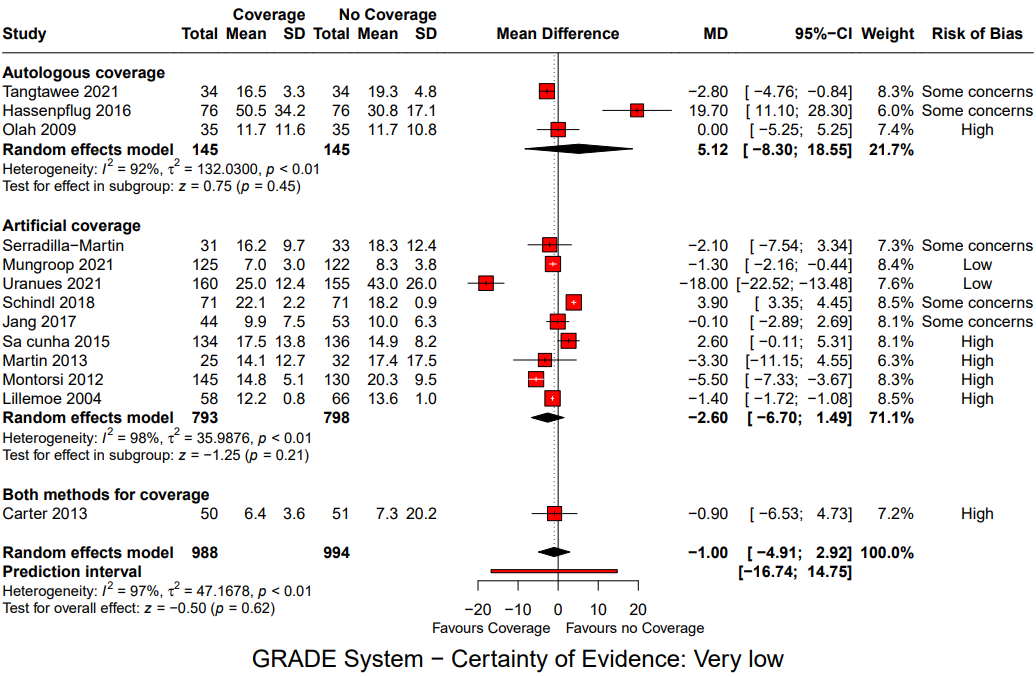** |

**Reasons for downgrading:** RoB -1 level, Inconsistency -1 level, Imprecision -1 level

| **Figure 42 Forest Plot of all studies for length of hospital stay according to type of operation** |
| --- |
| **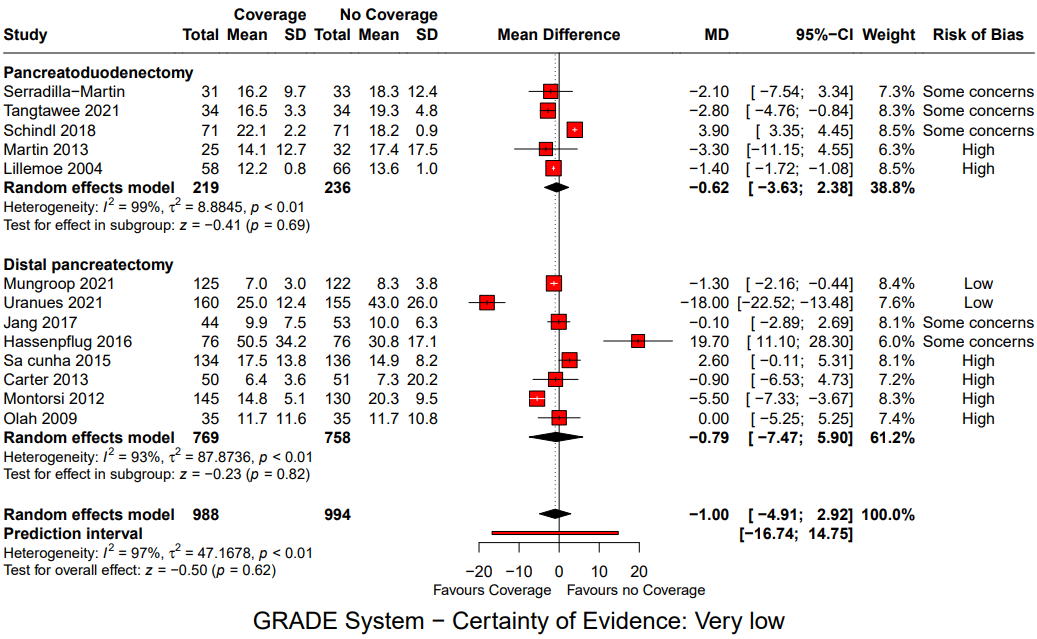** |

**Reasons for downgrading:** RoB -1 level, Inconsistency -1 level, Imprecision -1 level

| **Figure 43 Forest Plot of studies with artificial coverage and length of hospital stay** |
| --- |
| **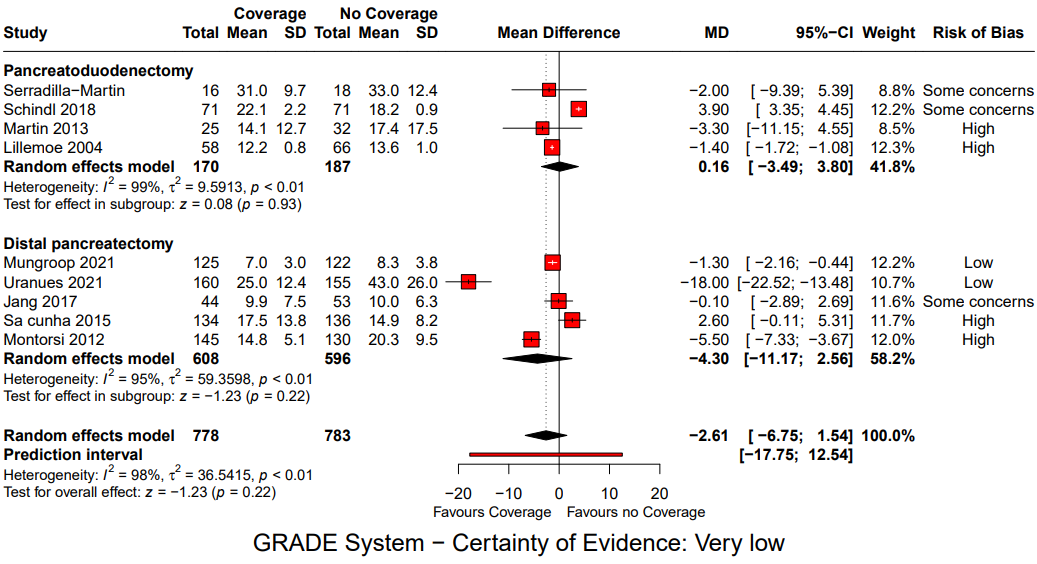** |

**Reasons for downgrading:** RoB -1 level, Inconsistency -1 level, Imprecision -1 level

**Figure 44 Forest Plot of studies with autologous coverage and length of hospital stay**

**
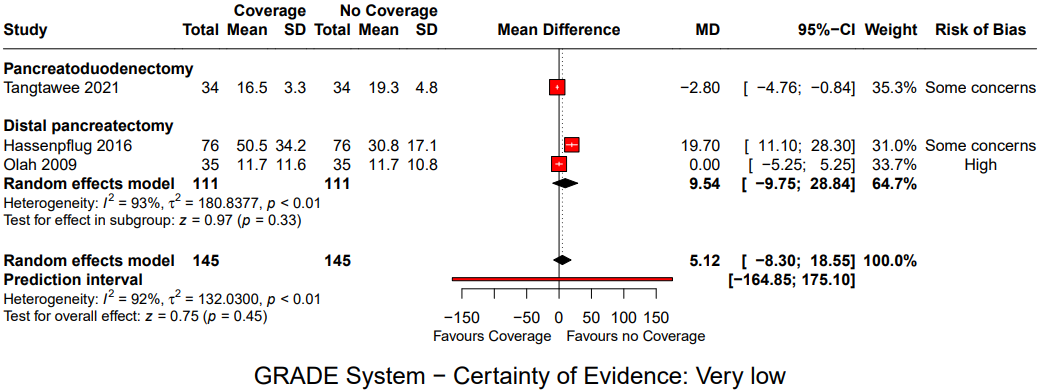
**

**Reasons for downgrading:** RoB -1 level, Inconsistency -1 level, Imprecision -1 level
